# Pyruvate Dehydrogenase Contributes to Drug Resistance of Lung Cancer Cells Through Epithelial Mesenchymal Transition

**DOI:** 10.3389/fcell.2021.738916

**Published:** 2022-01-04

**Authors:** Buse Cevatemre, Engin Ulukaya, Egemen Dere, Sukru Dilege, Ceyda Acilan

**Affiliations:** ^1^ Research Center for Translational Medicine, Koc University, Istanbul, Turkey; ^2^ Department of Biology, Uludag University, Bursa, Turkey; ^3^ Department of Clinical Biochemistry, Istinye University Faculty of Medicine, Istanbul, Turkey; ^4^ School of Medicine, Koc University, Istanbul, Turkey

**Keywords:** epithelial mesenchymal transition, lung cancer, drug resistance, pyruvate dehydrogenase complex, cancer metabolism

## Abstract

Recently, there has been a growing interest on the role of mitochondria in metastatic cascade. Several reports have shown the preferential utilization of glycolytic pathway instead of mitochondrial respiration for energy production and the pyruvate dehydrogenase (PDH) has been considered to be a contributor to this switch in some cancers. Since epithelial mesenchymal transition (EMT) is proposed to be one of the significant mediators of metastasis, the molecular connections between cancer cell metabolism and EMT may reveal underlying mechanisms and improve our understanding on metastasis. In order to explore a potential role for PDH inhibition on EMT and associated drug resistance, we took both pharmacological and genetic approaches, and selectively inhibited or knocked down PDHA1 by using Cpi613 and shPDHA1, respectively. We found that both approaches triggered morphological changes and characteristics of EMT (increase in mesenchymal markers). This change was accompanied by enhanced wound healing and an increase in migration. Interestingly, cells were more resistant to many of the clinically used chemotherapeutics following PDH inhibition or PDHA1 knockdown. Furthermore, the TGF*β*RI (known as a major inducer of the EMT) inhibitor (SB-431542) together with the PDHi, was effective in reversing EMT. In conclusion, interfering with PDH induced EMT, and more importantly resulted in chemoresistance. Therefore, our study demonstrates the need for careful consideration of PDH-targeting approaches in cancer treatment.

## 1 Introduction

The survival and growth of cancer cells usually involve changes in the metabolic pathways and reprogramming, in various cancer types, therefore dysregulated metabolism is considered as an important hallmark (Hanahan and Weinberg, 2011). Cancer cells have unique metabolic properties and there is a growing interest in elucidating the relationship between abnormal metabolic pathways and the progressive behaviors of cancer cells (Wei et al., 2020). The most well-described phenomenon is the Warburg effect (also known as aerobic glycolysis), in which glucose utilization becomes a priority for cancer cells even in the presence of sufficient oxygen (Warburg, 1956a; Warburg, 1956b). This unique aerobic glycolysis might provide several selective advantages, including resistance to apoptosis and therapeutic strategies, acidification of the tumor microenvironment and thus increased tumor invasion and metastasis ability (Gatenby and Gillies, 2004).

The pyruvate dehydrogenase (PDH), the gatekeeper enzyme in glucose metabolism, produces acetyl-CoA by oxidatively decarboxylating pyruvate to fuel mitochondrial tricarboxylic acid cycle (TCA). It plays a critical role in the metabolic axis by separating pyruvate between glycolysis and the TCA. Inhibition of PDH in cancer cells promotes the Warburg effect, and confers aggressive properties to cells (McFate et al., 2008; Luo et al., 2019). Decarboxylation of pyruvate is considered to be the rate-limiting step and catalyzed by the pyruvate dehydrogenase E1 (PDHA1) which is the catalytic component of PDH (Rajagopalan et al., 2015). E1 is a heterotetramer of two *α* and two *β* subunits and the active site containing-E1*α* is encoded by the *PDHA1* gene (Patel and Korotchkina, 2001). PDHA1 dysregulation has been implicated in metabolic reprogramming (Liu F. et al., 2015; Li et al., 2016a; Liu et al., 2018; Liu et al., 2019). Decreased expression of PDHA1 was found to be an unfavorable prognostic factor in various types of cancer including ovarian (Li et al., 2016b), gastric (Liu et al., 2018), prostate (Zhong et al., 2017), lung (Cao et al., 2017), liver (Sun et al., 2019), and esophageal cancer (Zhong et al., 2015). Furthermore, knockout of PDHA1 resulted in higher stemness phenotype in prostate cancer (Zhong et al., 2017) and led to Warburg effect with aggressive traits in esophageal squamous cancer *in vitro* and *in vivo* (Liu et al., 2019).

PDHA1 activity can be inhibited by the pyruvate dehydrogenase kinases (PDKs). Not surprisingly, it has been reported that the upregulation of PDK activity may contribute, in part, to the Warburg effect, resulting in inactivation of PDH (Kim et al., 2007). In a study of Zhang et al. (2019), PDK1 was found to be elevated in cisplatin-resistant cells (ovarian cancer) compared to cisplatin naïve-parental cells. Silencing PDK1 in resistant cells resulted in increased sensitivity against cisplatin, reversal of epithelial mesenchymal transition (EMT) and decreased cell motility, suggesting that the enhanced aerobic glycolysis not only drives tumor growth, but also provides metastatic advantage to cancer cells by EMT.

EMT is an important process with a predominant role in multiple conditions (physiological and pathophysiological) and known to be associated with drug resistance (Shibue and Weinberg., 2017). The oncogenic EMT process enables epithelial cells to acquire mesenchymal characters (through up- and down-regulation of mesenchymal and epithelial markers, respectively), thus providing cancer cells with increased invasive and migratory capacities, and resistance to chemotherapy. Indeed, these features are important drivers of cancer progression (Derynck and Weinberg, 2019). EMT is considered to be a key process for the initiation of metastatic cascade by allowing epithelial cancer cells to invade the extracellular matrix, enter the blood stream and eventually grow in a different tissue. In this cascade, a continuous nutritional supplement is required, which is provided by blood flow and metabolic reprogramming of cells (Sciacovelli and Frezza, 2017). Currently, there is accumulating evidence supporting that transcription factors that play an essential role in the EMT process also have regulatory functions in metabolic reprogramming (Kang et al., 2019). Metastasis is responsible for 90% of cancer-related mortalities (Chaffer and Weinberg, 2011), reinforcing the significance of blocking EMT. Therefore, providing knowledge on metabolic players in this process might be exploited for improved cancer therapy.

Although a number of studies have highlighted the significance of PDH activity or PDHA1 to promote metabolism and growth in cancer cells, the relationship between PDH and EMT has not been elucidated. Specifically, we hypothesized that PDH inhibition or PDHA1 knockdown might play a role in EMT and chemoresistance and investigated how disruption of PDH activity influenced the molecular events triggering these changes using lung cancer cells as a model.

## 2 Materials and Methods

### 2.1 Cell Culture

A549 cells were maintained in RPMI-1640 (Gibco, 21875-034) supplemented with 10% fetal bovine serum (Gibco, 10270-106) and 1% penicillin/streptomycin (Gibco, 15140-122). Cells were grown at 37°C in a humidified atmosphere with 5% CO_2_.

### 2.2 Knockdown of PDHA1 Using shRNA

PDHA1 human shRNA lentiviral vector plasmid and the corresponding control backbone (PDHA1 Human shRNA Plasmid Kit, TL310532) was obtained from OriGene Technologies. Lentivirus was produced by co-transfecting the lentiviral vector, the packaging plasmid (PAX2), and the envelope plasmid (pMD2.G) into HEK-293T cells using Lipofectamine™ 3000 (Thermo Fisher Scientific, L3000015). Supernatants were collected 48 h after transfection and filtered through a 0.45 μm membrane. A549 cells were transduced with lentiviral supernatant (with 8 μg/ml polybrene, Sigma, TR-1003-G) and then selected with 2 μg/mL puromycin (Sigma, 540411) to obtain cells with stable knockdown of PDHA1.

### 2.3 Cell Proliferation Analysis

#### 2.3.1 Sulforhodamine B (SRB) Assay

Cells were seeded on 96-well plates (3.5 × 10^3^ per well) the day prior to drug exposure. At the end of the treatment, cells were fixed by using trichloroacetic acid (10%, Sigma, T6399) at 4°C for 1 h. Plates were washed with ddH_2_O and allowed to dry at room temperature (RT). SRB (0.4%, Santa Cruz, sc-253615A) was used to stain the cells for 30 min and subsequently washed with 1% acetic acid (Sigma, 100063). The bound SRB dye was solubilized by using Tris base (10 mM, BioShop, TRS001). The optical density was measured at 564 nm with a microplate reader (FLASH Scan S12, Analytical Jena).

#### 2.3.2 ATP Assay

Cell were seeded and treated as depicted in 2.3.1. ATP assay was performed as described previously (Karakas et al., 2015) and the content of ATP was measured by using a luminometer with a measuring time of 1 s (FLx800, BioTek). The cell viability for both SRB (Abs) and ATP (RLU) assays was calculated by using the formula:

$$\%\text{Viability}=100\times {\text{Treatment}}_{\left(\text{Abs}/\text{RLU}\right)}/{\text{Control}}_{\left(\text{Abs}/\text{RLU}\right)}$$

#### 2.3.3 xCELLigence Real Time Cell Analyzer (RTCA)

Cells were seeded on E-plates (3.5 × 10^3^ per well, Roche) the day prior to drug exposure. Cell growth was monitored at 1 h intervals for 72–96 h by using xCELLigence real time cell analyzer (Roche). Cell index (CI) graphs were generated by using the RTCA software of the manufacturer.

#### 2.3.4 Colony Formation Assay

A549 cells were seeded on 6-well plates (1× 10^3^) and cultured for additional 15 days. Culture media was changed every 3 days. After this period, cells were washed with PBS and fixed by using ice cold methanol at −20°C for 10 min. Crystal violet (0.05%, Sigma, C6158) was used to stain the cells for 15 min and subsequently washed twice with ddH_2_O. Images of the colonies were taken by using an Olympus CKX41 inverted microscope.

### 2.4 Wound Healing

A549 cells were seeded on 12-well plates (1.2 × 10^5^ per well) and after a day cells were wounded by scratching with a 10 μL tip. Wells were rinsed with PBS and fresh culture medium containing Cpi613/Devimistat (BioVision, 9464) was added on cells. The movement of untreated and treated (6.25–25 μM) cells in the wounded area were photographed by using an inverted microscope (Olympus CKX41) at 0, 12, 24, and 30 h.

### 2.5 Transwell Invasion Assay

Transwell assay was performed using the cell culture inserts (pore size 8 μm, BD353097). Briefly, inserts were coated with matrigel (0.4 mg/ml, Corning, 356234). After incubating at 37°C overnight, the Matrigel solidified and coated inserts were placed on 24-well plates. Harvested cells were seeded on the upper well in 1% FBS containing media and the lower wells were filled with complete media. After 24 h, insides of the inserts were brushed with wet cotton swab to remove non-invaded cells and invaded cells on the outside were fixed by using methanol, stained with crystal violet (0.05%). Bound violet as solubilized with acetic acid (10%) and the optical density was measured at 570 nm with a microplate reader (FLASH Scan S12, Analytical Jena).

### 2.6 Phalloidin Staining

A549 cells were seeded (1 × 10^4^) on 8-well chamber slides (Corning, 354630). A day after cells were fixed by using 4% formaldehyde (Thermo Fisher Scientific, 28906) for 15 min at RT, permeabilized with 0.1% Triton X-100 for 10 min, and blocked with 3% BSA (Cell Signaling Technology, 9998) for 1 h. DyLight™ 554 phalloidin (Cell Signaling Technology, 13054) was used to stain F-actin (1:200 dilution in PBS) for 15 min at RT. Chamber slides then incubated with Prolong Gold Antifade Mountant containing DAPI (Thermo Fisher Scientific, P36931).

### 2.7 Annexin-V Staining

A549 cells were seeded on 6-well plates (1 × 10^5^) the day prior to Cpi613 exposure (24, 48 and 72 h). After the treatment period, cells were detached by trypsinization and stained by using Annexin V/7-aminoactinomycin D at RT in the dark for 20 min (Annexin V & Dead Cell kit, Luminex, MCH100105). The percentage of apoptotic cells was calculated by Muse™ Cell Analyzer (Luminex).

### 2.8 qRT-PCR

RNA samples were extracted from cells using Total RNA Purification Kit (Jena Bioscience, PP-210L) and cDNA was synthesized from 500 ng total RNA using the SCRIPT cDNA Synthesis Kit (Jena Bioscience, PCR-511S). Subsequently 10 ng cDNA template was amplified using qPCR GreenMaster with UNG (Jena Bioscience, PCR-369L). *β*-Actin was used as reference control and real-time quantitative PCR was run on LightCycler 480 (Roche). The relative fold change in gene expressions were measured with the 2^(−ΔΔCT)^ method.

### 2.9 SDS-PAGE and Western Blotting

Total proteins were extracted by using RIPA lysis buffer (Santa Cruz, sc-24948) containing protease inhibitor cocktail and sodium orthovanadate. PMSF was added at a final concentration of 2 mM (Sigma, P7626). Cells were lysed on ice for 30 min and subsequently the lysate was centrifuged to separate proteins (4°C, 15 min, 13.000 g). The resulting supernatant was quantified using BCA assay (Thermo Fisher Scientific, 23227). Samples were boiled (95°C, 7 min) after mixing with NuPAGE LDS sample buffer (Thermo Fisher Scientific, NP0007) and NuPAGE Sample reducing agent (Thermo Fisher Scientific, NP0004). Twenty μg of total protein were subjected to 12% SDS-PAGE (150 V, 50 min) and transferred onto a nitrocellulose membrane using the iBlot (Thermo Fisher Scientific, IB301001). After blocking the membranes with 5% non-fat dry milk (BioShop, SKI400) at RT for 1 h, primary antibodies (1:1000) were applied and incubated at 4°C overnight. Antibodies against e-cadherin (3195S), vimentin (5741S), actin (4970S), n-cadherin (13116), *α*-sma (14968S), mdrp-1 (72202S), mdr-1 (13978S), histone H3 (9715S), *α*-tubulin (2144S), p44/42-MAPK (4695), p38-MAPK (9212), p-c-jun (9164S), and glut1 (12939) were from Cell Signaling Technology and pdha1 (ab67592) from Abcam. Following washes in TBST, membranes were incubated with HRP-linked secondary antibody (1:2000, Cell Signaling Technology, 7074) at RT for 1 h and probed with Immobilon Forte Western HRP substrate (Merck, WBLUF0100), and imaged by Fusion FX-7 imaging system (Vilber Lourmat, France).

## 3 Results

### 3.1 PDHi Inhibits the Growth of Cancer Cells

**FIGURE 1:**
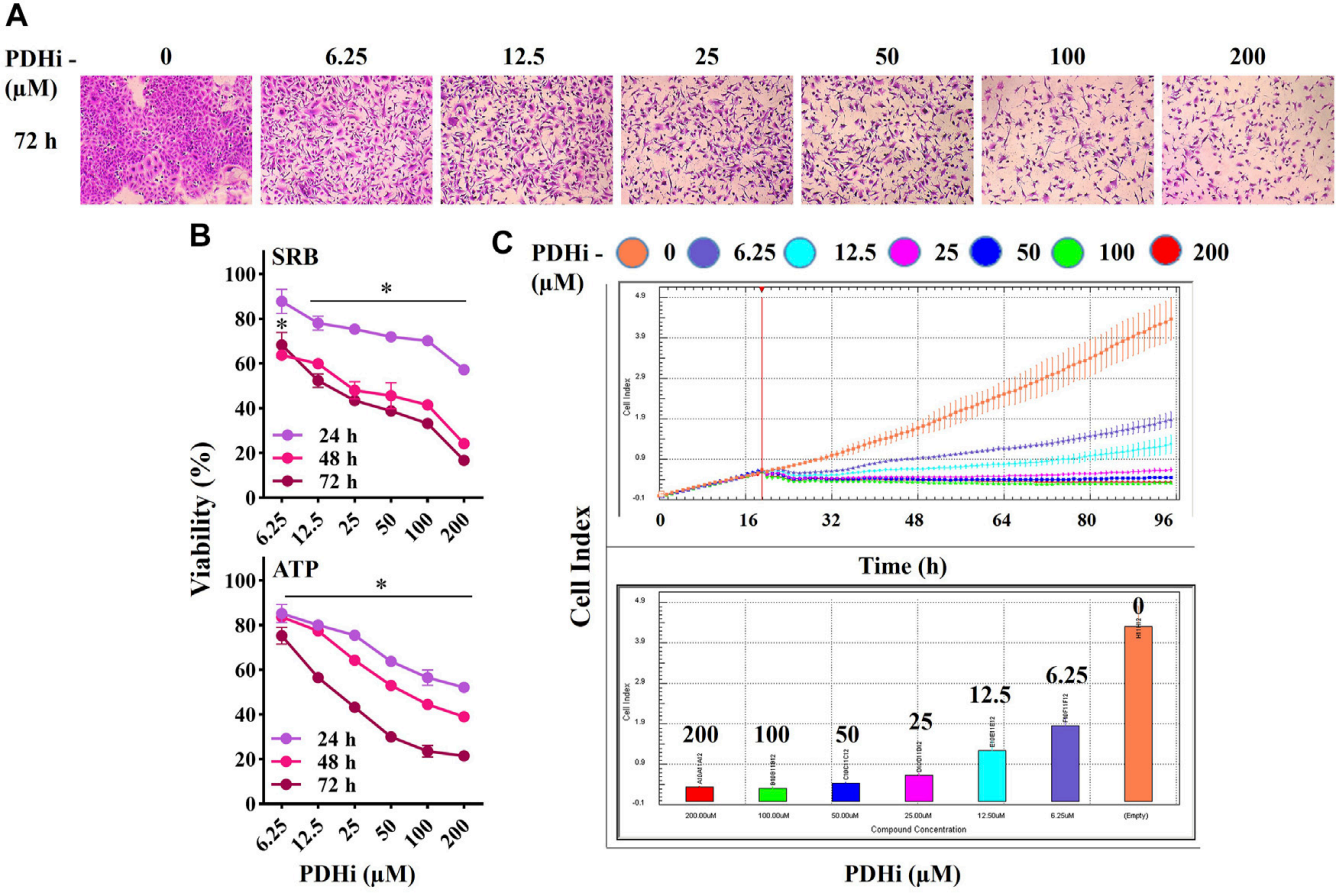
PDHi treatment resulted in growth inhibition. **(A)** A549 cells were treated (72 h) with PDHi at indicated doses and after staining with SRB dye, cells were photographed under phase contrast microscope (magnification 100×). **(B)** The effect of PDHi on the growth of A549 cells was quantified by SRB and ATP viability assays. Data are presented as mean ± SEM. **(C)** The cell index (CI) values for 0–200 μM (represented by different colours) of PDHi on A549 cells was generated by real time cell analyzer (RTCA). * Significantly difference compared to untreated cells (*p* < 0.0001).

In order to examine the effect of PDHi (Cpi613/Devimistat) on cancer cell growth, its cytotoxicity was evaluated on a number of cells of different origins such as lung (A549), breast (MCF7) and colorectal (HT29) cancers. The viability of each cell line was significantly decreased in a time (24, 48 and 72 h) and concentration dependent (6.25–200 µM) manner in response to PDHi exposure (Figure 1) (Supplementary Figures S1–S3). The IC_50_ values at 72 h (based on ATP assay) were 18.6, 99.2, and 54.5 μM against A549, MCF7, and HT29, respectively. To differentiate PDHi-induced cytotoxic, cytostatic and antiproliferative effects, real time cell monitoring was used for all of the cells. While lower doses (25–100 µM) were mostly cytostatic, higher doses (200 µM) led to cytotoxicity in all cell lines tested (Figure 1C; Supplementary Figures S2C, S3C). The most responsive of these lines (A549) was chosen for further functional studies. Indeed, PDHi-induced growth inhibition correlated with apoptosis induction as evidenced by DNA fragmentation, Annexin-V staining, and rescue of cell death *via* pretreatment with z-VAD-FMK, a pan-caspase inhibitor, in A549 cells (Supplementary Figure S4). Based on these observations, sublethal doses of PDHi (6.25, 12.5 and 25 µM) were chosen to analyze EMT-related phenotypes for further studies. At these selected doses of PDHi, flow cytometric analysis revealed G_2_/M-phase cell accumulation in A549 cells, suggesting a mechanism for the growth suppressive effect of PDHi (Supplementary Figures S5A–C).

### 3.2 PDHi Induces Epithelial Mesenchymal Transition-Like Phenotype in A549 Lung Cancer Cells

**FIGURE 2:**
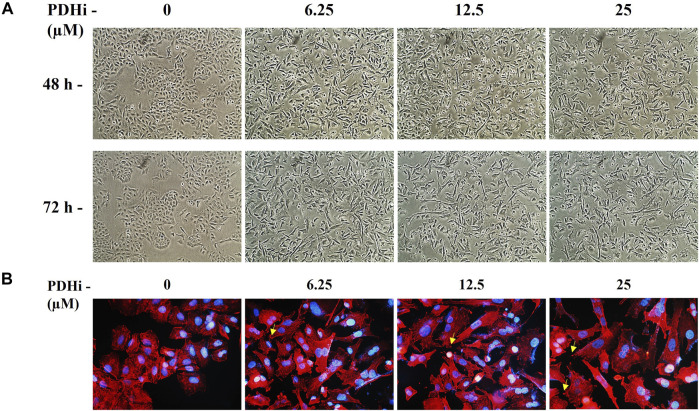
PDHi-induced morphological changes in A549 cancer cells. **(A)** Cells treated with PDHi for 48–72 h (Supplementary Figure S6 for morphological changes in HT29 and MCF7 cancer cells) at indicated doses and were photographed under phase contrast microscope (magnification 100×). **(B)**. Phalloidin staining indicated F-actin (red) reorganization in PDHi treated A549 cells. DAPI was used to stain DNA (blue, magnification 400×).

**FIGURE 3:**
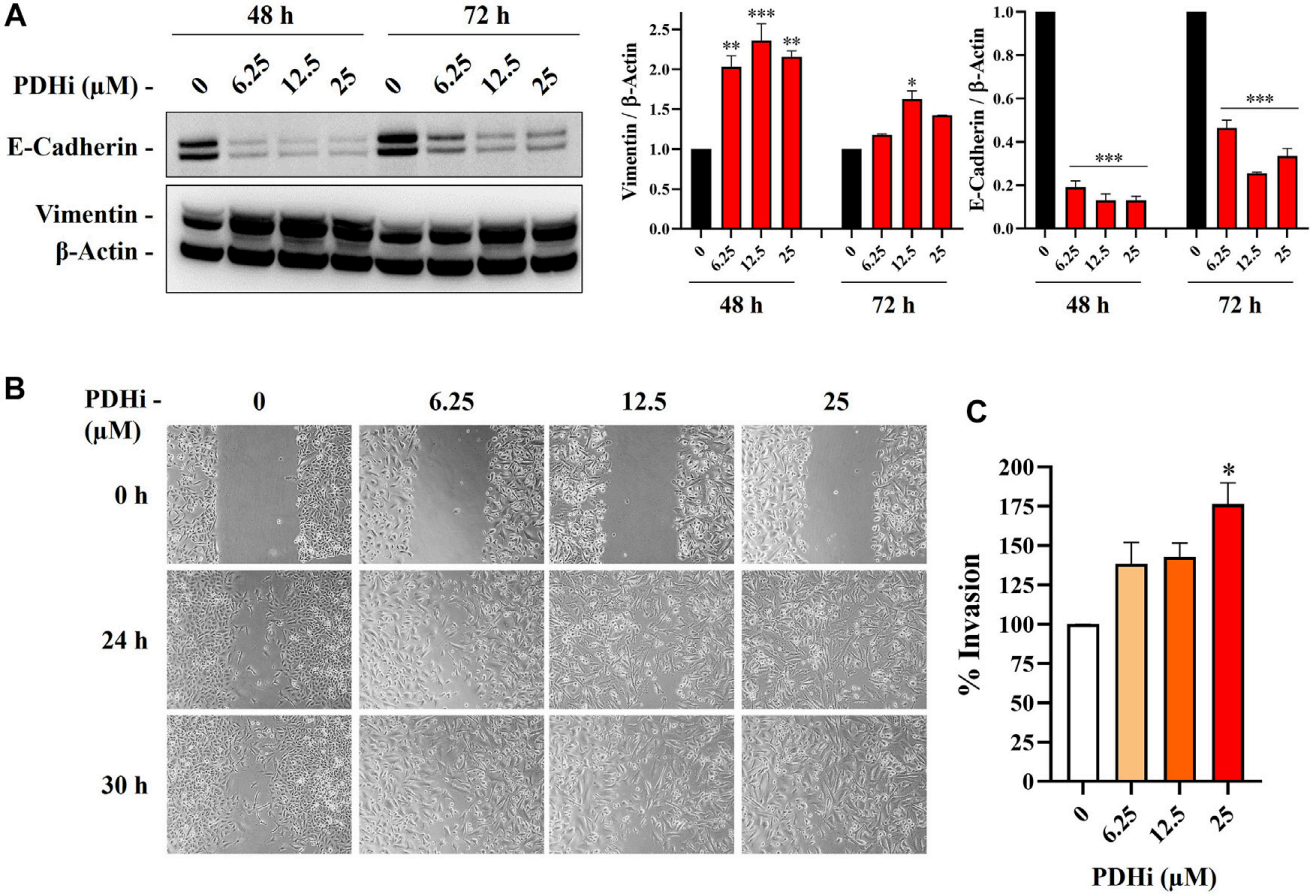
PDHi led to the induction of EMT phenotype. **(A)** Western blot analysis showing the decrease of E-cadherin (epithelial marker) and increase in vimentin (mesenchymal marker) in PDHi treated A549 cells. **(B)** Wound healing assay was performed in A549 cells when cultured with PDHi at indicated doses and time points. The results are shown at 100× magnification. **(C)** Transwell invasion assay was conducted on PDHi treated (24 h) A549 cells. The invaded cells were stained and counted. *Significantly difference compared to untreated cells (*p < 0.01, **p < 0.001, ***p < 0.0001).

To explore the effect of PDHi on cell morphology, PDHi-treated A549, HT29, MCF7 cells were monitored using phase contrast microscopy. Interestingly, while untreated cells grew in tight clumps and have the cobblestone appearance that is a characteristic of epithelial cells, inhibition of PDH resulted in elongated/irregular fibroblastic appearance, suggesting mesenchymal-like phenotypes may have been triggered in response to PDHi (Figure 2; Supplementary Figure S6). Since cells need to acquire additional cell-matrix contact points during EMT, the remodeling of actin cytoskeleton was evaluated following inhibition of PDH *via* phalloidin staining (Figure 2B). As expected, untreated epithelial cells were organized in cortical thin bundles. In contrast, PDHi treatment induced bundling of actin filaments into thick and elongated contractile stress fibers at the cell attachment sites, as typically seen in transdifferentiated mesenchymal cells (Figure 2B). Therefore, our morphological observations led us to inquire whether other molecular or biological changes associated with EMT could also be observed following PDH inhibition. In order to pursue this aim, we investigated the expressions of e-cadherin and vimentin (epithelial and mesenchymal markers, respectively) using western blotting. Consistent with morphological observations, inhibition of PDH resulted in increased vimentin expression and decreased e-cadherin expression, supporting induction of EMT-like phenotypes (Figure 3A; Supplementary Figure S7). We determined the expressions of kinases (PDK1-4) that play an important role in the regulation of the PDH complex. Among these inactivating kinases, we found that expression of PDK4 was significantly increased (at all the time points tested, 6–24 h) in a dose-dependent manner (Supplementary Figure S8). High expression of PDK4 can have both oncogenic or tumor suppressive properties, and has been previously associated with EMT, albeit with controversial results (Atas et al., 2020). Our findings support an oncogenic role for PDK4 in response to PDH suppression. Indeed, more work should be performed to determine the exact molecular mechanisms as to how these cellular events are linked.

It has been well demonstrated that EMT confers migratory and metastatic properties to cancer cells (Yang and Weinberg, 2008). Therefore, we performed wound healing and invasion assays to investigate the ability of lung cancer cells to migrate/invade after they were subjected to different concentrations of PDHi. As expected, greater migration was observed in PDHi-treated cell to untreated cells in wound healing migration assays (Figure 3B). PDHi-treated A549 cells showed approximately 2-fold increase in invasion compared to untreated cells (Figure 3C). These results demonstrate that the PDHi-induced EMT phenotype significantly accelerates cancer cell migration and invasion *in vitro*.

### 3.3 TGF-*β* Signaling May Be Involved in PDHi-Induced Epithelial Mesenchymal Transition

**FIGURE 4:**
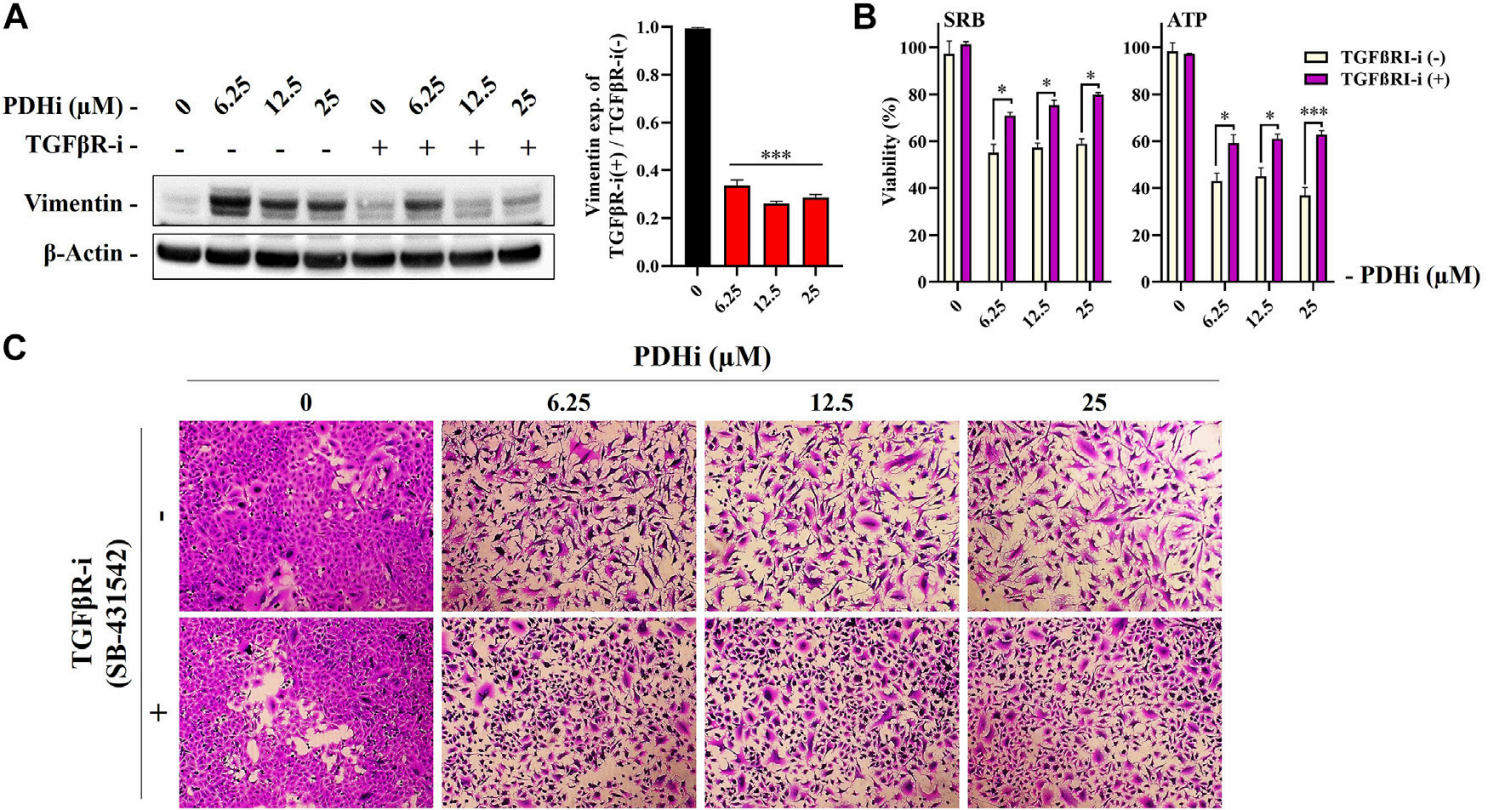
Treatment with a TGF*β*RI inhibitor (SB-431542) reversed PDHi-induced vimentin expression and morphological changes. The cells were co-incubated with 2.5 µM of TGF*β*RI-i and PDHi for 72 h. **(A)** Treated cells were analyzed by western blot for the expression levels of Vimentin and *β*-Actin was used as a loading control. Data are presented as mean ± SEM. *Significantly difference compared to PDHi-alone (***p < 0.0001) **(B)** Rescue of growth inhibition was analyzed by SRB and ATP viability assays. Data are presented as mean ± SEM. *Significantly difference compared to PDHi-alone (*p < 0.01, ***p < 0.0001). **(C)** Morphology of PDHi and/or TGF*β*RI-i treated cells for 72 h and cells were visualized by using the SRB dye (magnification 100×).

Among the cues in the extracellular microenvironment, transforming growth factor *β* (TGF*β*) is known to be a prominent inducer of EMT through cellular pathways involving several transcription factors. To understand whether TGF*β* pathway is important for the EMT related changes seen in response to PDHi, we used an inhibitor targeting TGF*β*RI, SB-431542 (TGF*β*RI-i) and determined whether PDHi-induced EMT can be reversed *via* TGF*β* inhibition. Supporting involvement of the TGF*β* pathway, addition of TGF*β*RI-i was sufficient to significantly reduce vimentin expression (Figure 4A). Next, we then examined the ability of TGF*β*RI-i to block the PDHi-mediated inhibition of cell proliferation. PDHi efficiently suppressed the growth of A549 cells, whereas TGF*β*RI-i significantly blocked PDHi-induced growth inhibition as determined by both SRB and ATP cell viability assays (Figure 4B). Furthermore, TGF*β*RI-i reverted PDHi-induced elongated/irregular fibroblastic morphology to an epithelial-like morphology (Figure 4C).

We hypothesized that if PDHA1 inhibition leads to drug resistance *via* triggering EMT, suppressing EMT may result in reversion of the drug resistance. Since TGF*β* is a well-known inducer of EMT, we used RepSox (TGF*β*RI) to suppress it. shPDHA1-induced cells were subjected to cisplatin in combination with RepSox and then cell death was measured to determine whether cells were more sensitive to cisplatin. Consistent with our model, PDH inhibition did not lead to cisplatin resistance when EMT was inhibited *via* TGF*β* inhibitor (Supplementary Figure S9).

This data prompted us to consider whether there is a PDH-related regulation when EMT is stimulated. For this purpose, we induced EMT in A549 cells by applying TGF*β*I and then determined the expression levels of PDH genes (namely, PDHA1, PDHB, and PDHX) by qRT-PCR. Interestingly, we observed a reduction in transcript levels in these genes upon TGF*β*I treatment (Supplementary Figure S10). Therefore, apparently there is two sided feedback between PDH and EMT. However, it is not clear whether this is a direct effect or through other molecular players.

Furthermore, among the SMAD proteins of the canonical TGF*β* signaling pathway, we looked at the expressions of SMAD2, 3, and 4 following PDH inhibition. We found that only SMAD4 expression decreased in a dose- and time-dependent manner (Supplementary Figure S11). Consistent with our data, previous research has shown that SMAD4 reduction is associated with phenotypes such as tumor progression and metastasis in some cancers including lung cancer (Fukuchi et al., 2002; He et al., 2011; Tan et al., 2021), therefore is supportive with our findings of PDH inhibition leading to a more aggressive phenotype.

### 3.4 PDHi Confers Resistance to Chemotherapeutic Drug-Induced Cell Death

**FIGURE 5:**
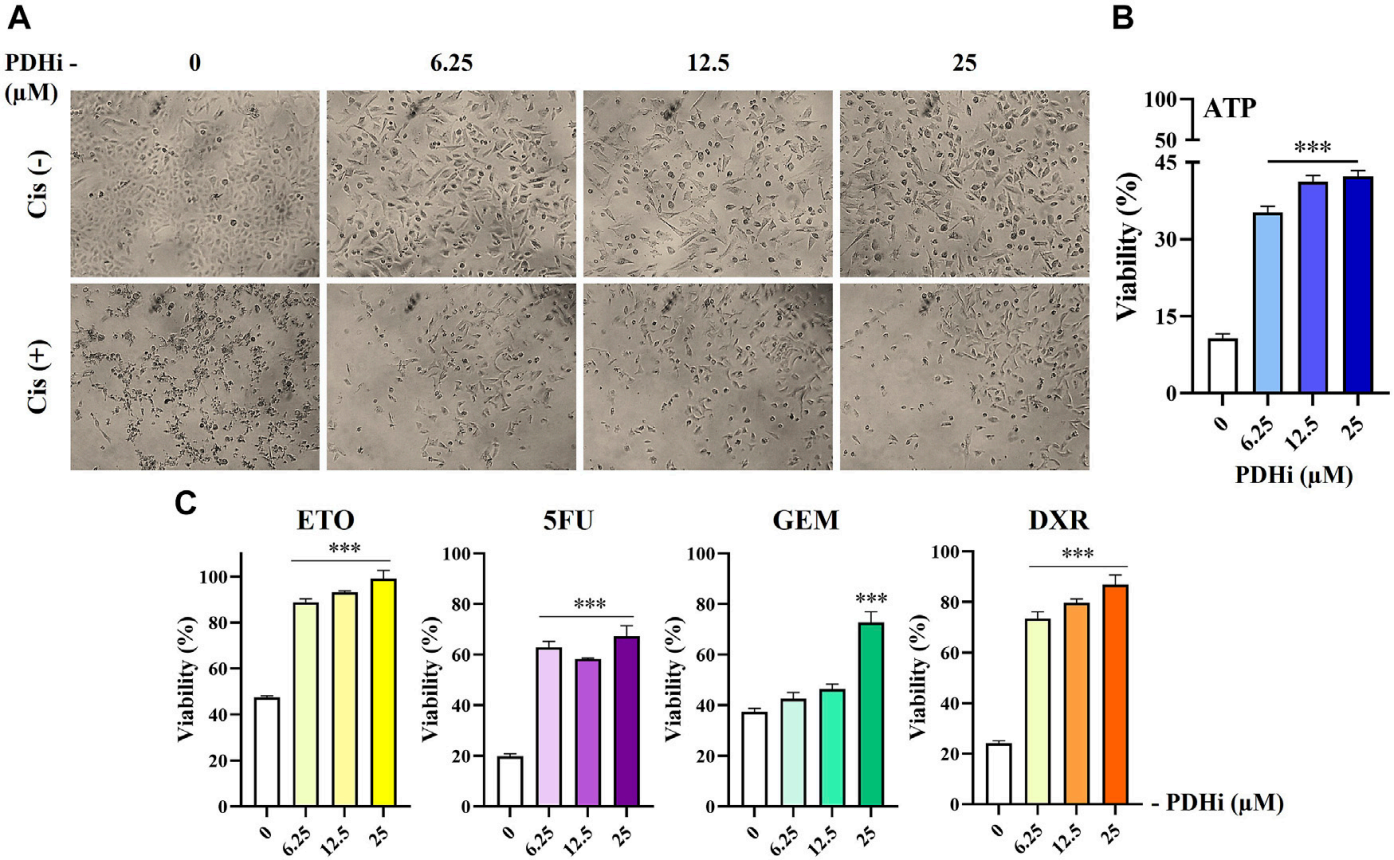
PDHi treatment decreased the chemotherapeutic drug sensitivity of A549 cells. **(A–C)** PDHi pretreated (6.25, 12, 25 µM) or non-pretreated (0 µM) A549 cells were exposed to Cisplatin (25 µM) for 72 h, and **(B,C)** the cell viability was examined by ATP assay. Data are presented as mean ± SEM. *Significantly difference compared to 0 μM, one-way ANOVA with subsequent Tukey test (***p < 0.0001).

Since the acquisition of EMT phenotype generally correlates with chemoresistance, we measured the survival of PDHi-pretreated cells in response to treatment with commonly used chemotherapeutic drugs. Consistent with EMT-like changes, inhibition of PDH increased resistance of cancer cells to all of the drugs tested in this study, while cell viability was drastically reduced in non-treated cells upon treatment with the cytotoxic agents (cisplatin, doxorubicin, etoposide, 5-FU, and gemcitabine) (Figure 5; Supplementary Figure S12). Since the mechanism of action of these drugs are different from each other, this finding suggested a more general mechanism for cellular protection such as increase in survival signals or inhibition of apoptotic pathways, which will be examined further.

### 3.5 Knockdown of PDHA1 Induces Epithelial Mesenchymal Transition-Like Morphological Changes

**FIGURE 6:**
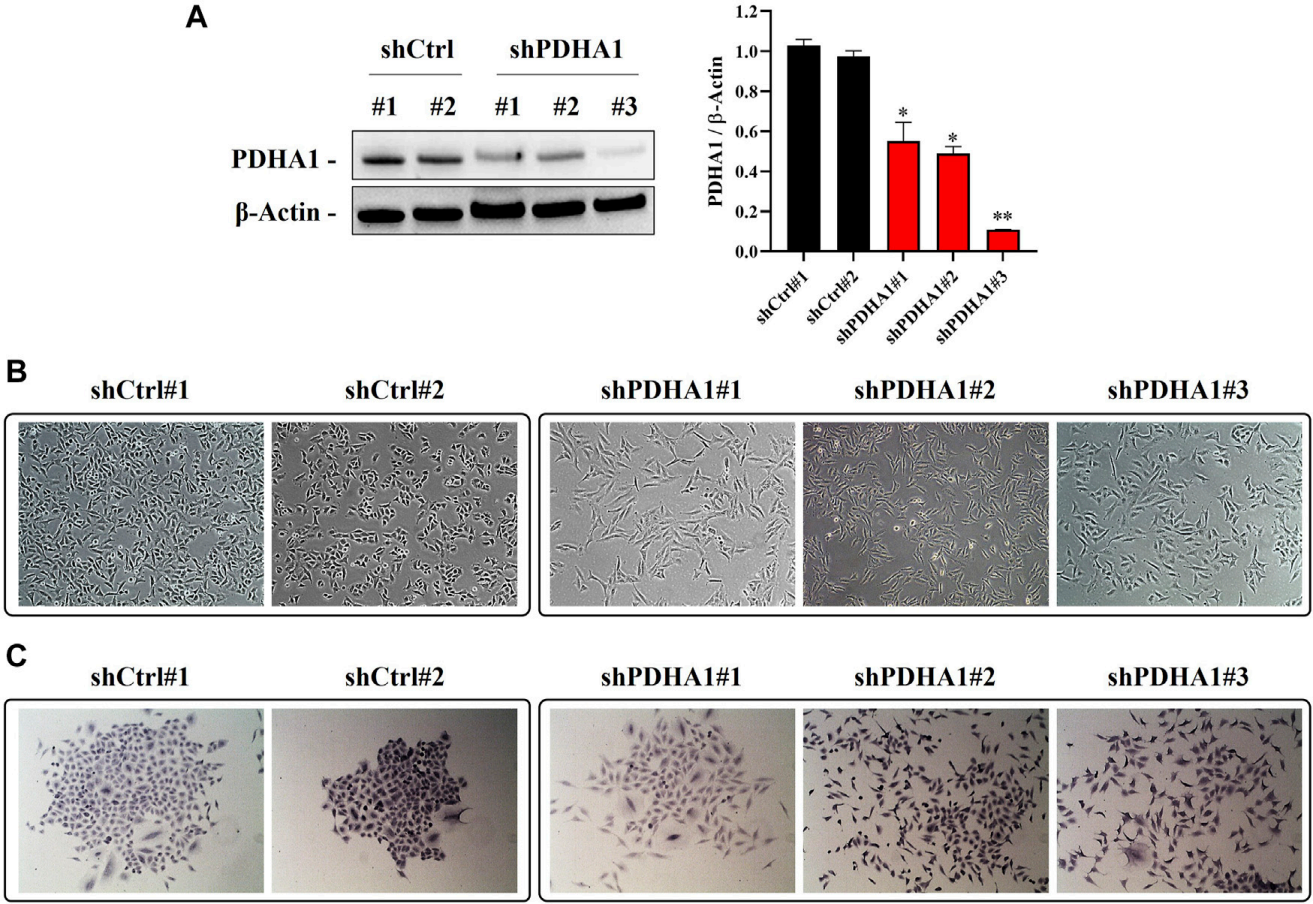
PDHA1-silenced lung cancer cells exhibited morphological changes. **(A)** Knockdown (stable) efficiency of shPDHA1 and the control (shCtrl) vectors in A549 lung cancer cells was determined *via* western blotting to confirm PDHA1 protein expression. Data are presented as mean ± SEM. *Significantly difference compared to shCtrl cells (*p < 0.01, **p < 0.001) **(B)** ShCtrl cells showed an epithelioid, cobblestone rounded appearance. On the contrary, the phenotypic changes in shPDHA1 cells includes loss of cell polarity and a spindle-like morphology (magnification 100×). **(C)** shPDHA1 cells showed scattered morphology as compared with the control backbone transfected shCtrl cells (magnification 200×).

To explore whether observed effect is directly a result of PDH inhibition or a consequence of some off-target phenotype, we established a number of PDHA1 knockdown clones derived from A549 cells. We validated our cells by showing the significant reduction in protein expression levels of PDHA1 in shPDHA1 group of A549 cells compared with the control (shCtrl) cells (Figure 6A). When grown to confluence, shPDHA1 cells did neither form a monolayer nor exhibited epithelial, cobblestone-like morphology as seen in shCtrl cells, but rather displayed a spindle-shaped mesenchymal phenotype and acquired a spread morphology (Figures 6B,C). The effect of PDHA1 silencing on lung cancer cell growth/proliferation was also explored by performing doubling assays. As indicated in Supplementary Figure S13, the doubling of cells was significantly slower in PDHA1 knockdown group compared with those in the shCtrl group.

**FIGURE 7:**
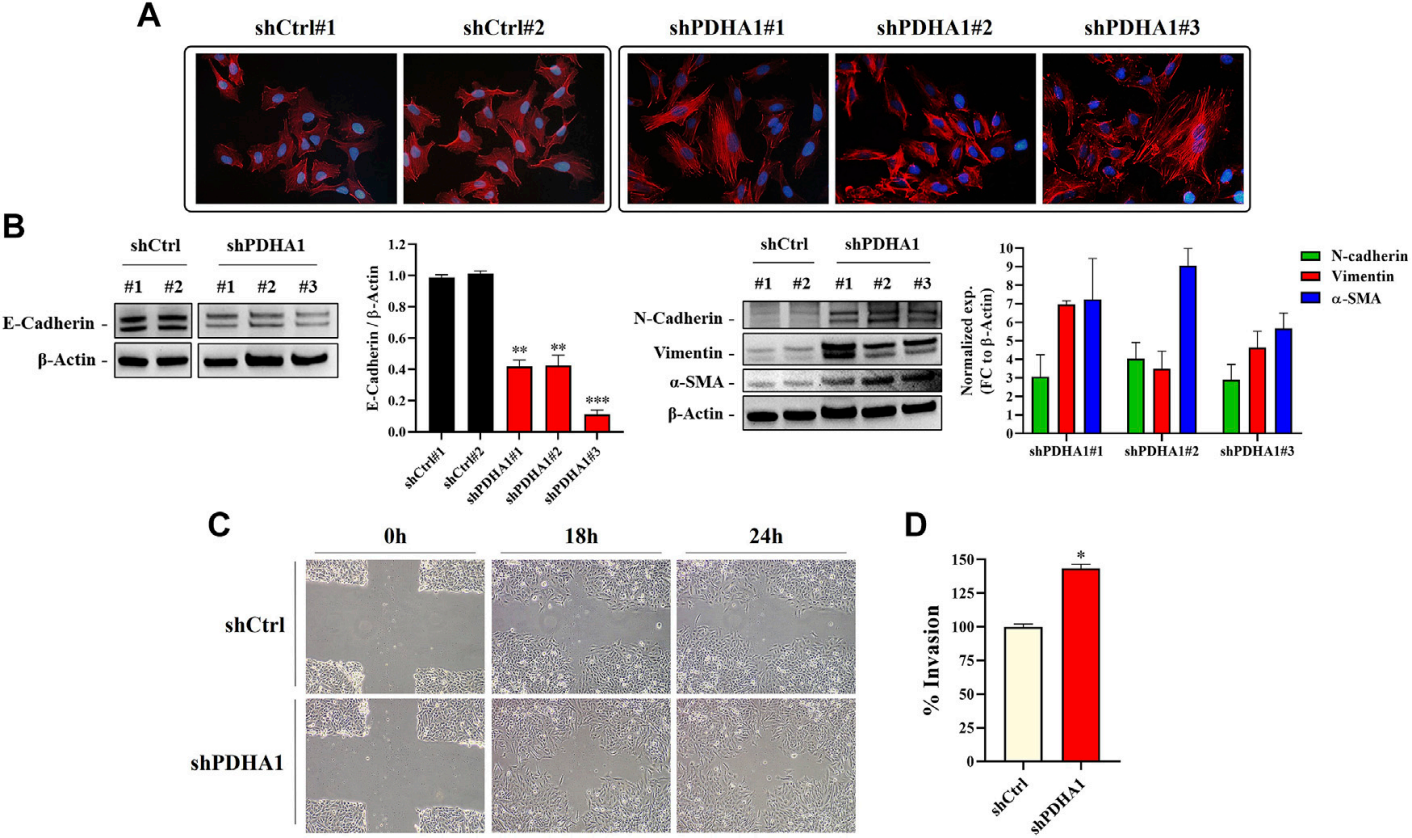
PDHA1 knockdown induced EMT features in human lung cancer cells. **(A)** Phalloidin staining indicated F-actin (red) reorganization in shPDHA1 and shCtrl cells. DAPI was used to stain DNA (blue, magnification 400×). **(B)** Western blot analysis showing the loss of E-cadherin (epithelial marker) and the increase in N-cadherin, vimentin, and *α*-SMA (mesenchymal markers) in shPDHA1 cells. *Significantly difference compared to shCtrl cells (**p < 0.001, ***p < 0.0001) **(C)** Wound healing assay was performed in shPDHA1 and shCtrl cells. The results are shown at 100× magnification. **(D)** Transwell invasion assay was conducted on shPDHA1 and shCtrl cells for 24 h. The invaded cells were stained and counted. *Significantly difference compared to shCtrl cells (*p < 0.01).

To confirm if the cytoskeletal changes observed after treatment with the PDHi is also valid following silencing of PDHA1, the stress fibers resulted from F-actin reorganization was examined by phalloidin staining. As seen before, shCtrl cells showed cortical actin staining, whereas the shPDHA1 cells exhibited elongated F-actin stress fibers (Figure 7A). Similar to PDHi, expression of e-cadherin was found to be downregulated, whereas mesenchymal proteins, n-cadherin, vimentin and *α*-SMA, were upregulated after PDHA1 knockdown as compared to shCtrl cells (Figure 7B). Once again, a greater migration capacity was observed in PDHA1 knockdown cells as compared to shCtrl cells in wound healing assay (Figure 7C). Furthermore, transwell invasion assay showed increased number of invading cells following PDHA1 silencing (approximately 1.5 fold) compared to that of shCtrl cells (Figure 7D). Cumulatively, these results favor phenotypes associated with EMT in response to inhibition or silencing of PDHA1 activity.

### 3.6 Knockdown of PDHA1 Expression Confers Chemoresistance

**FIGURE 8:**
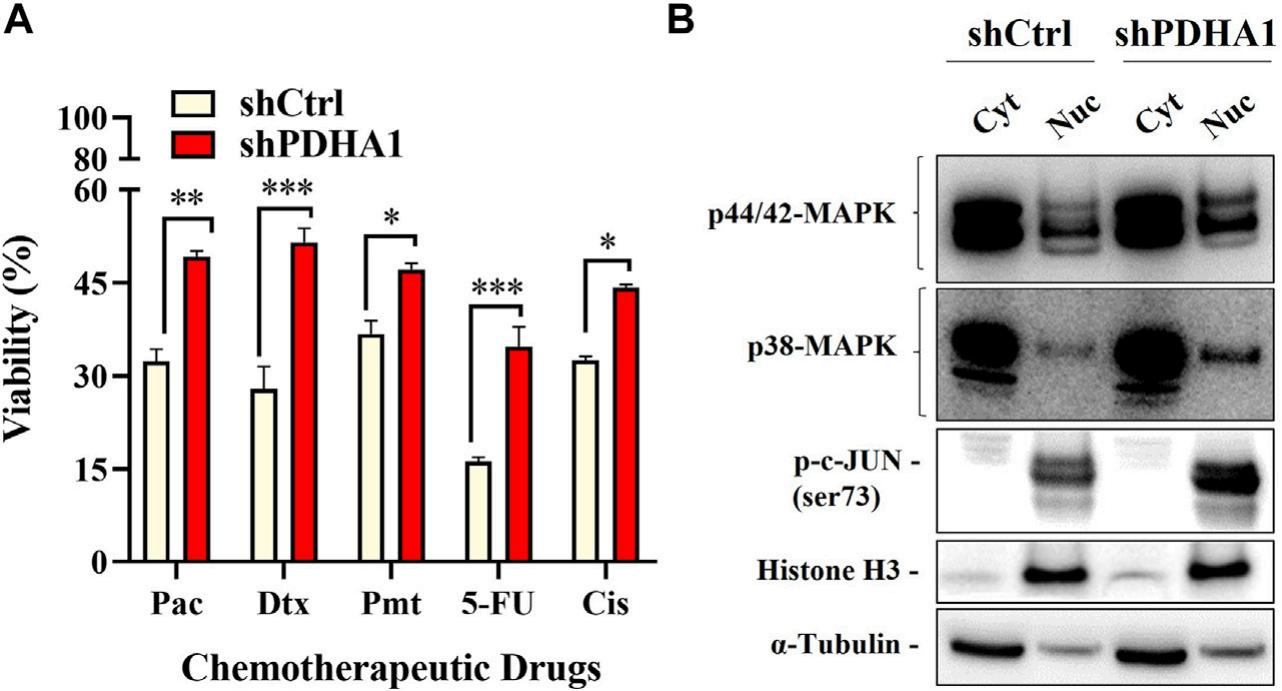
PDHA1 knockdown induced chemoresistance in lung cancer cells. **(A)** shPDHA1 cells are more chemoresistant than shCtrl cells at individual doses of paclitaxel, docetaxel, pemetrexed, and 5FU, as shown by ATP viability assay. *Significantly difference compared to shCtrl cells (*p < 0.05, **p < 0.001, ***p < 0.0001) **(B)** p44/42 MAPK (Erk1/2), p38-MAPK, p-c-JUN proteins in shCtrl and shPDHA1 cells were determined by western blotting.

We next elucidated whether PDHA1 accounts for chemoresistance. For this purpose, ATP viability assay was used to evaluate the chemoresistance between shCtrl and shPDHA1 cells. Cells were exposed to chemotherapeutic drugs (paclitaxel, docetaxel, pemetrexed, and 5FU) that are commonly used for the treatment for lung cancer. The results demonstrated that cells treated with shPDHA1 displayed significantly better survival ability than shCtrl cells (Figure 8A)**.**

Aberrant activation of signaling pathways of Erk MAPK and p38 MAPK was shown to be essential for drug resistance, EMT and growth of cancer cells (Li et al., 2016c). Abnormalities in MAPK signaling have been implicated to be required in cancer development and progression (Dhillon et al., 2007). To identify which signaling pathway was mediated, the phosphorylation levels of p44/42 MAPK (Erk1/2) and p38 were examined in shPDHA1 cells. Interestingly, the phosphorylation levels of these proteins were elevated remarkably in PDHA1 knockdown cells, suggesting that in addition to increase in drug efflux, proliferative signals are also stimulated in response to inhibition of PDHA1 (Figure 8B).

## 4 Discussion

The goal of this study was to investigate whether PDH, the gate-keeper of mitochondria, is involved in EMT. Mitochondrial dysfunction is shown to be positively correlated with increased aggressiveness and metastatic potential in cancer (Lunetti et al., 2019). mtDNA depletion induced-mitochondrial dysfunction induced EMT in human mammary epithelial cells (MCF7 and MCF10A) (Guha et al., 2014). In support of this finding, downregulation of mitochondrial genes was found to be associated with poor clinical outcome across 20 different types of solid cancers (TCGA) and correlates with the expression of EMT-gene signature (Gaude and Frezza, 2016). Furthermore, EMT has been reported as a prominent phenotype in tumors harboring mutations in enzymes of the TCA cycle. For instance, accumulation of fumarate caused by the loss of fumarate hydratase (FH), resulted in EMT signatures (Sciacovelli et al., 2016). Similarly, silencing FH increased the clonogenicity and enhanced the migratory and invasive potentials of nasopharyngeal cancer cells (He et al., 2016). Cells deficient in succinate dehydrogenase (SDH), another enzyme that functions in TCA, displayed an EMT-associated metastatic phenotype (Loriot et al., 2015). The relationship between SDHB loss and EMT was also shown in colon (Wang et al., 2016) and ovarian cancer (Aspuria et al., 2014). The mutations in isocitrate dehydrogenase mutations can lead to the production and accumulation of produce 2-hydroxyglutarate (2-HG), which was shown to induce EMT. Grassian et al. (2012) reported a direct correlation between intracellular 2-HG levels and EMT characteristics. Citrate synthase (CS) is another TCA cycle enzyme that is associated with EMT. CS knockdown induced morphological changes consistent with EMT features in cervical cancer and resulted in enhanced metastasis and proliferation in *in vitro* and *in vivo* models. (Lin et al., 2012).

In accordance with these studies, we demonstrated that PDH inhibition or PDHA1 knockdown resulted in morphological and molecular changes (increase in the expression of vimentin and a decrease in the expression of E-cadherin) consistent with the mesenchymal phenotype. The present study also demonstrated that administration of PDHi may promote these EMT-like changes in A549 lung cancer cells, in part *via* the activation of TGF*β* signaling, as TGF*β*RI-i was sufficient to significantly reduce vimentin expression and revert PDHi-induced elongated/irregular fibroblastic morphology to an epithelial-like morphology. The observation of increased cell migration and invasion of PDHi-treated or shPDHA1 cells was also consistent with these results. Although the underlying mechanism is not clear, it is most likely due to the activation of TGF*β* pathway. In addition, these cells grew more slowly compared to the untreated/shCtrl cells. Mechanisms underlying EMT-induced drug resistance may include slow growth rate (Zhang and Weinberg, 2018). It was shown that slowly dividing pancreatic cell subpopulations show an expression profile consistent with CSC (cancer stem cell) and EMT signatures (Dembinski and Krauss, 2009). In another study, cell proliferation has been shown to be impaired when Snail (an EMT-inducing transcription factor) was expressed in cells (Vega et al., 2004). The reported Snail-driven mechanism includes the impairment of transition from early to late G_1_ by maintaining low levels of Cyclins D and the blockage of G_1_/S transition by maintaining high levels of p21. While the drug resistance phenotype observed in our study might be a result of EMT trigger through PDH inhibition, we cannot rule out the fact that G_2_/M accumulation (Supplementary Figure S5) could have also contributed to the resistance outcome.

It has been shown that EMT conferred to drug resistance of NSCLC (Thomson et al., 2005; Rho et al., 2009) and TGF*β* is a pivotal driver of these multistep process (Hua et al., 2020). Accordingly, we hypothesized whether PDH could contribute to the chemoresistance in cancer cells. Consistent with their EMT characteristics, PDHi or shPDHA1 treated cells showed less sensitivity to cisplatin (compared with untreated/shCtrl cells) and to other chemotherapeutic drugs used in this study. The previous reports and our observations together confirmed that the NSCLC with the EMT phenotype is prone to develop resistance to various drugs, including cisplatin (Wang et al., 2018). Although the underlying mechanism was not clear, our study has confirmed the relationship between drug resistance and EMT in A549 cells.

In cells, the mitogen-activated protein kinase (MAPK) signaling cascade allows cells to transduce extracellular signals into appropriate adaptive intracellular responses and this network is considered to be associated with EMT (Gui et al., 2012). In addition to Smad signaling pathways, TGF*β* can also activate a variety of non-Smad signaling pathways (Ras, MAPKs, PI3K/Akt) which are shown to be also associated with TGF*β*-induced EMT (Bakin et al., 2002; Xie et al., 2004; Tavares et al., 2006). In addition, studies have found Erk1/2 to be involved in tumour chemoresistance (Liu Q.-H. et al., 2015). Aberrant activation of Erk signaling (triggered by Erk2 amplification) was shown to be the underlying mechanism for acquired resistance against EGFR inhibitors in NSCLC (Ercan et al., 2012). Zheng et al. (2013) showed that gemcitabine resistant pancreatic cancer cells (Panc1) constitutively produced high levels of pERK1/2 (p44/42 MAPK). Likewise, when we manipulated PDHA1 in A549 cells, p44/42 and p38 signaling pathways were also triggered. With regard to whether PDHA1-induced EMT dependent on these pathways, subsequent research is necessary to confirm these findings.

In the study by Cao et al., 196 of NSCLC tissues and adjacent normal tissues were immunohistochemically detected and the correlation between PDHA1 expression and the pathological characteristics of NSCLC was examined. Their results suggested that expression of PDHA1 was related to differentiation degree of tissues, lymphatic metastasis and TNM staging. Specifically, the expression of PDHA1 protein in patients with high and intermediate differentiation was higher compared with low differentiated ones. Patients without lymphatic metastasis showed higher expression of PHDA1 protein than those with it. Additionally, patients in stage I and II had higher expression of PDHA1 protein compared to those in stage III and IV, suggesting that the lower expression of PDHA1 was associated with poor prognosis in NSCLC patients. In the same study, the overall survival rate was significantly different with a reported survival of 78.9% in the PDHA1-positive group, and 60.0% in the PDHA1-negative group.

**FIGURE 9:**
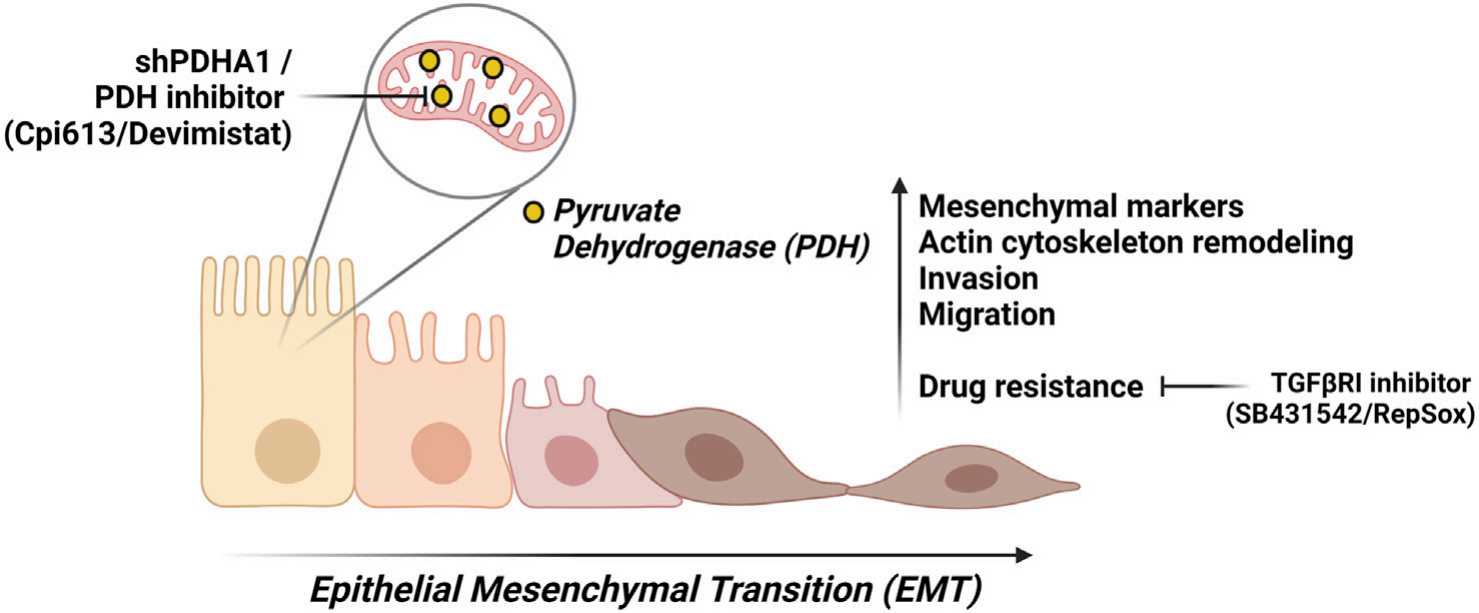
A summary of this study. We suggest that PDH inhibition either through chemical inhibitor (Devimistat) or shPDHA1, leads to EMT-like phenotypes in lung cancer cells. These phenotypes include an increase in migration capability and invasion. Furthermore, PDH inhibition confers to resistance to several chemotherapeutic drugs, and this can be reverted *via* inhibition TGF*β* pathway. Created with BioRender.

Our results suggest that PDHA1 may affect the lung cancer cell proliferation, invasion, migration, and chemoresistance, thereby affecting lung cancer progression. Although the precise role of PDHA1 in EMT process remains to be elucidated, the results from our study support that loss of PDHA1 or inhibition of PDH may act as an EMT-inducer. We cannot refer to a single molecule that is responsible for our observations. However, it is probably a cumulative series of events that lead to EMT upon PDH inhibition. Additionally, our finding proposes a mechanism by which cells treated with PDHi or shPDHA1 may acquire chemoresistance by undergoing EMT (Figure 9). Inhibiting or reversing the EMT process may cause re-sensitization of chemoresistant cells. Collectively, the above results suggest that the use of EMT-inhibitors in combination with PDHi’s could become a rational strategy for the treatment of solid tumors especially lung cancer.

## Data Availability

The original contributions presented in the study are included in the article/Supplementary Material, further inquiries can be directed to the corresponding author.
