# Supplementary material for: Pyruvate Dehydrogenase Contributes to Drug Resistance of Lung Cancer Cells Through Epithelial Mesenchymal Transition
Source: Front Cell Dev Biol. 2022 Jan 4;9:738916. doi: 10.3389/fcell.2021.738916 (PMC8785343; doi:10.3389/fcell.2021.738916)
Supplement: Supplementary file 1 [file DataSheet1.PDF]

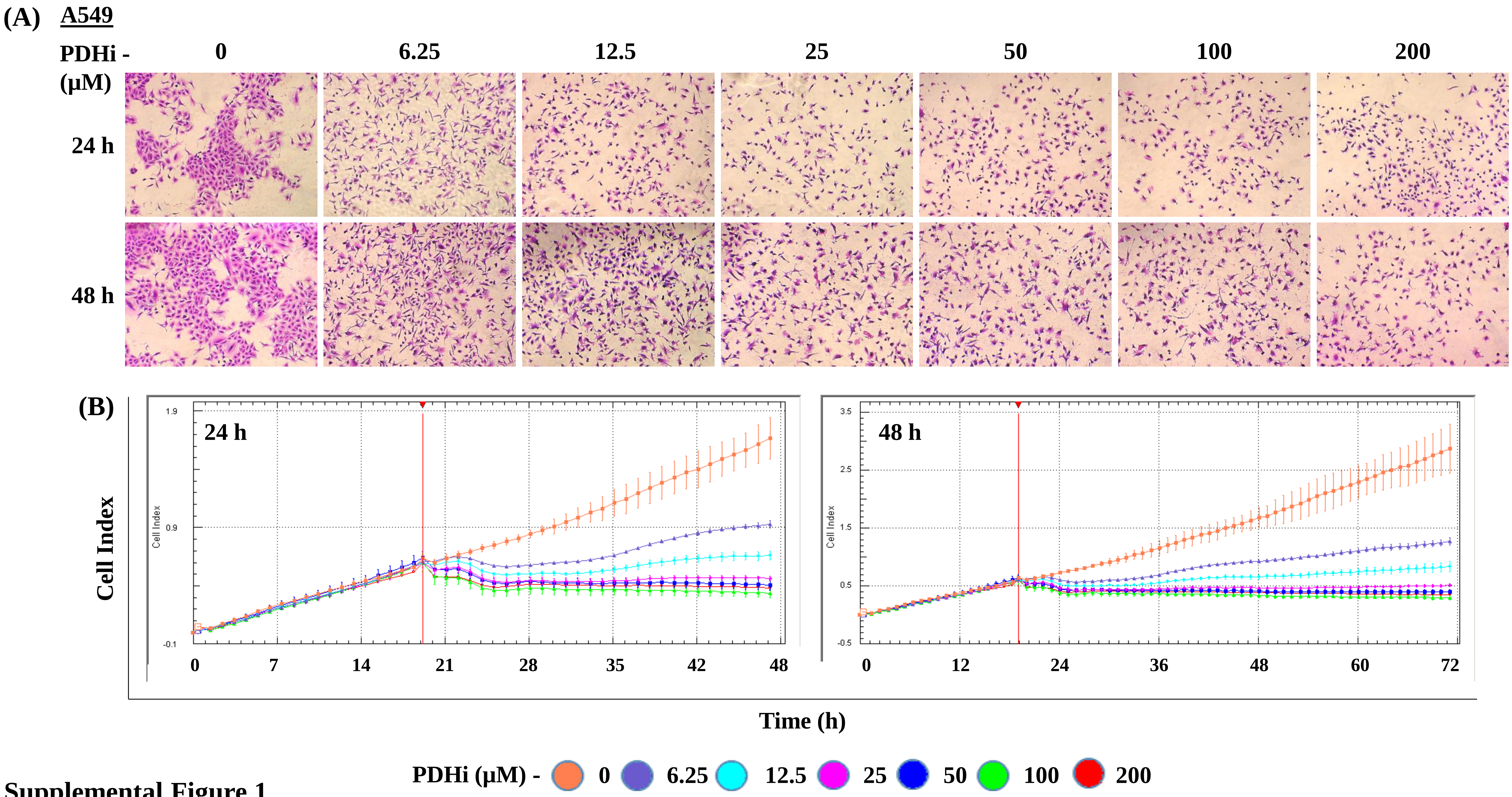

Supplemental Figure 1

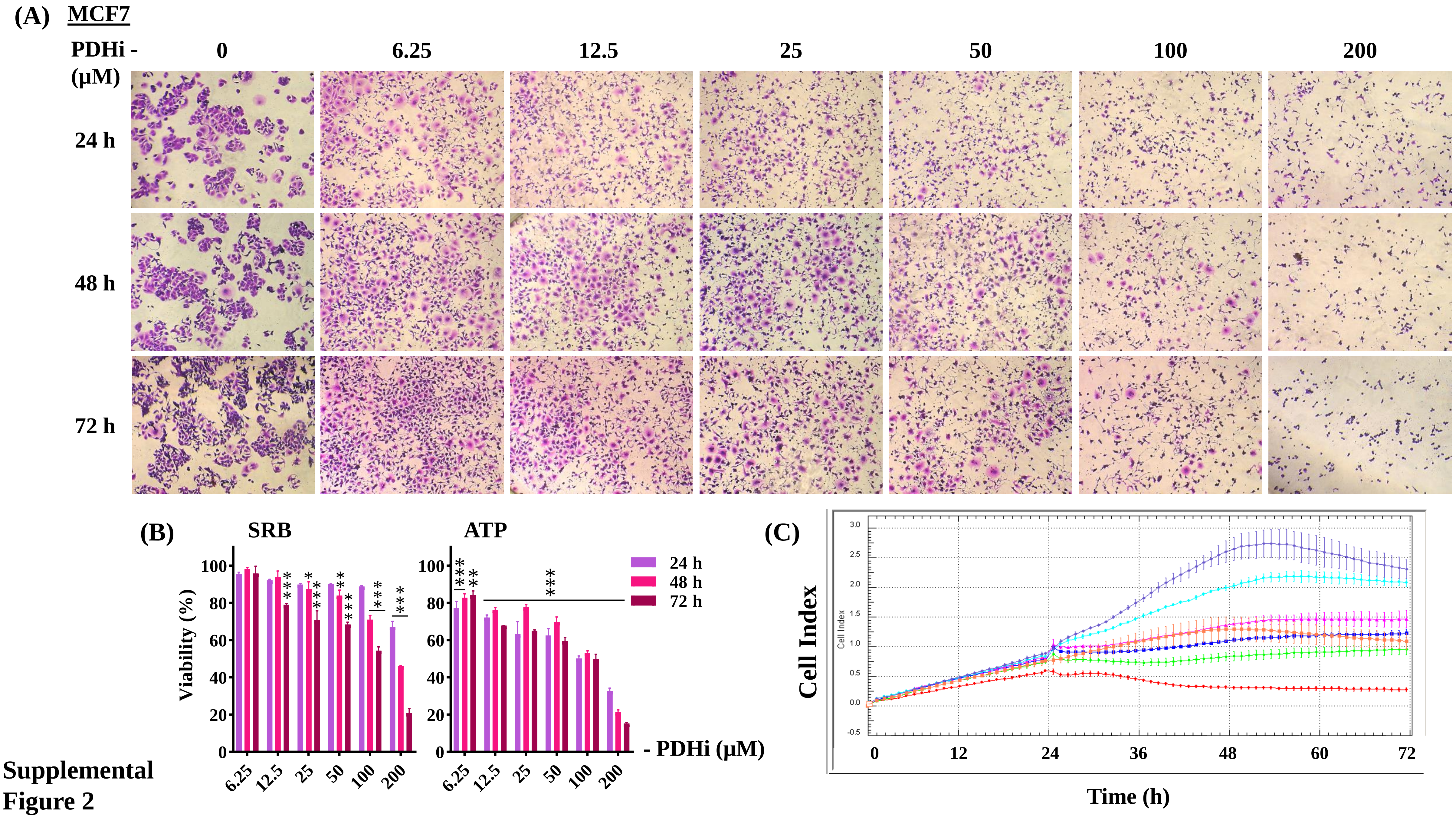

Supplemental Figure 2

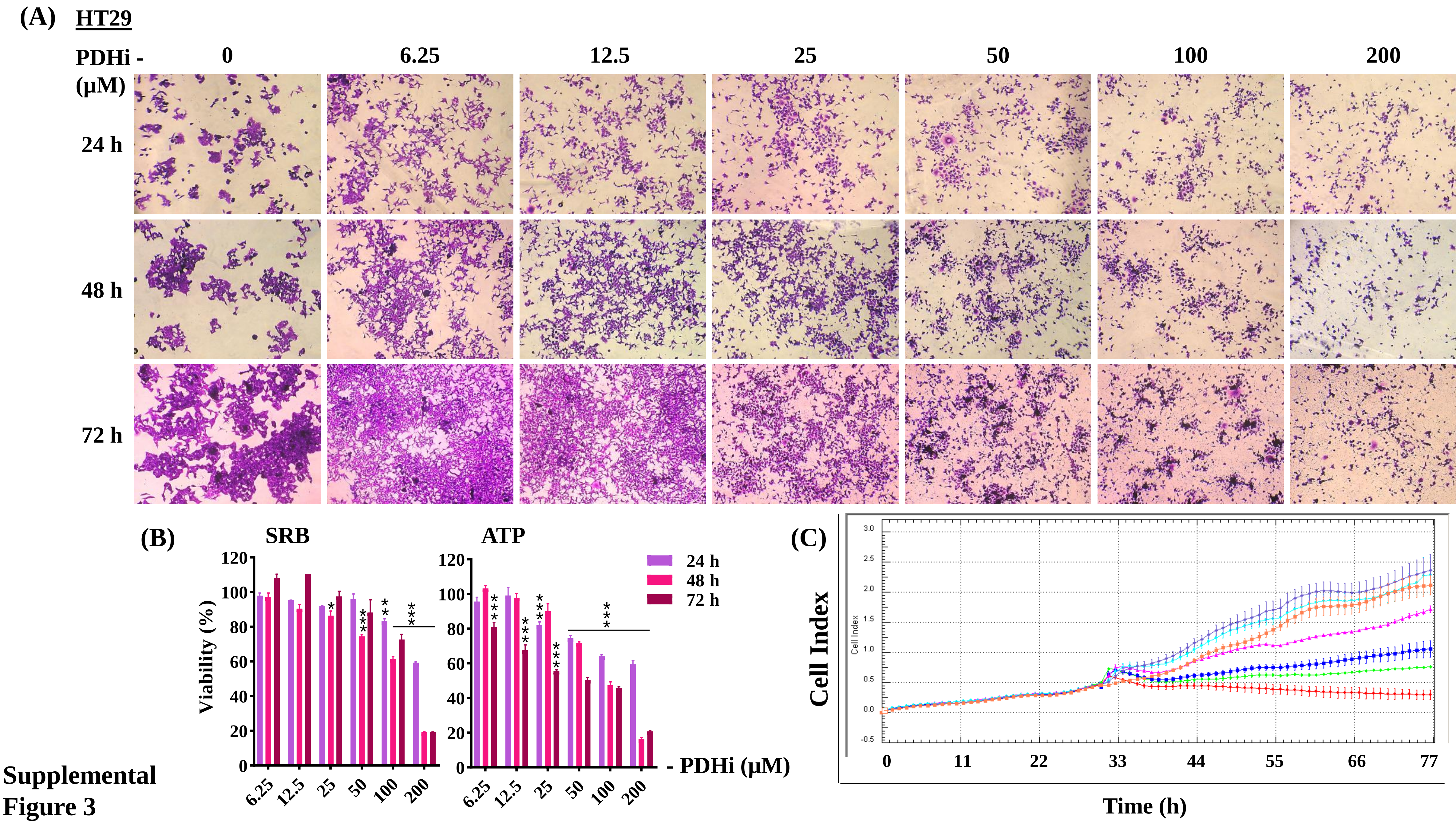

Supplemental Figure 3

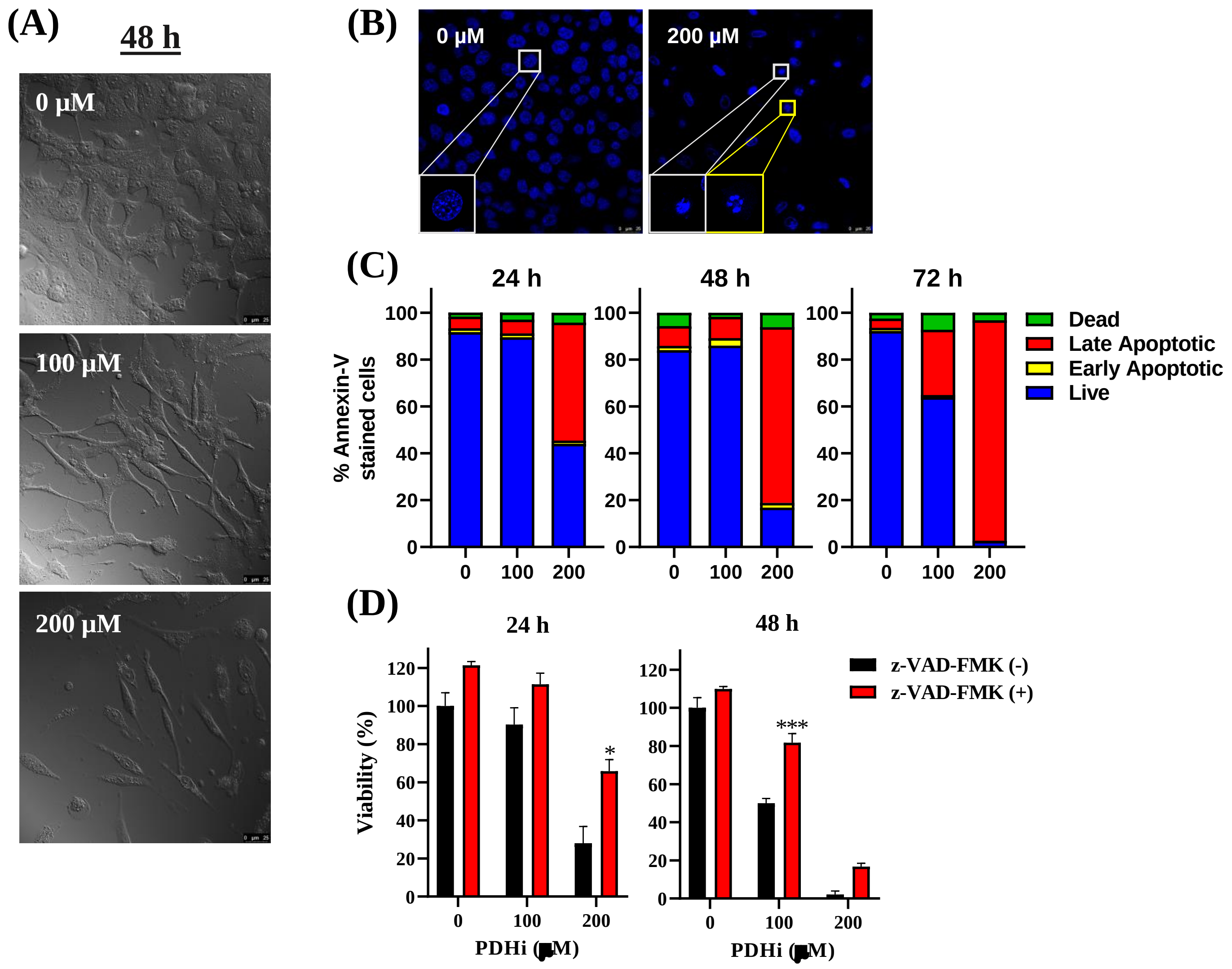

Supplemental Figure 4

(A) A549

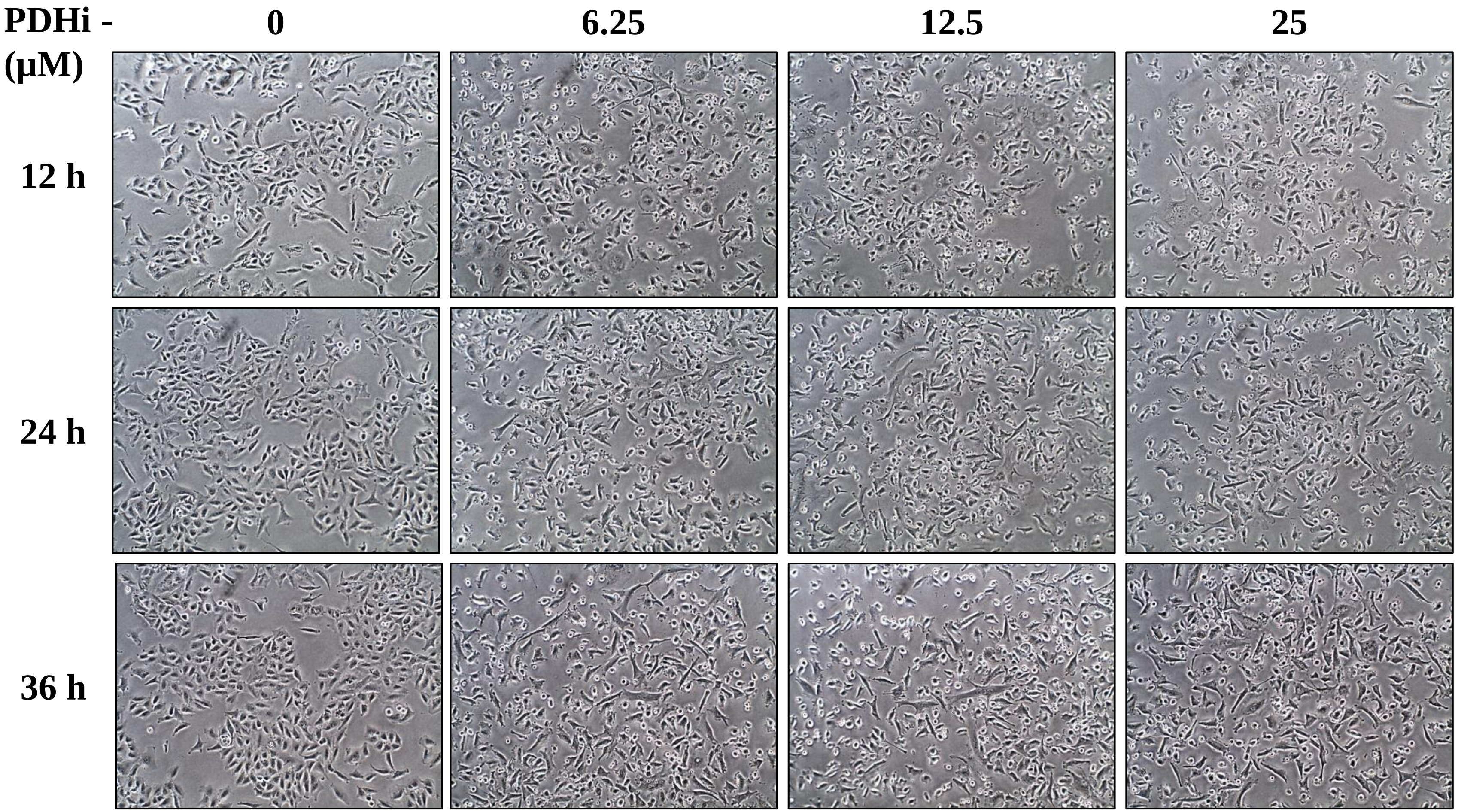

Supplemental Figure 5A

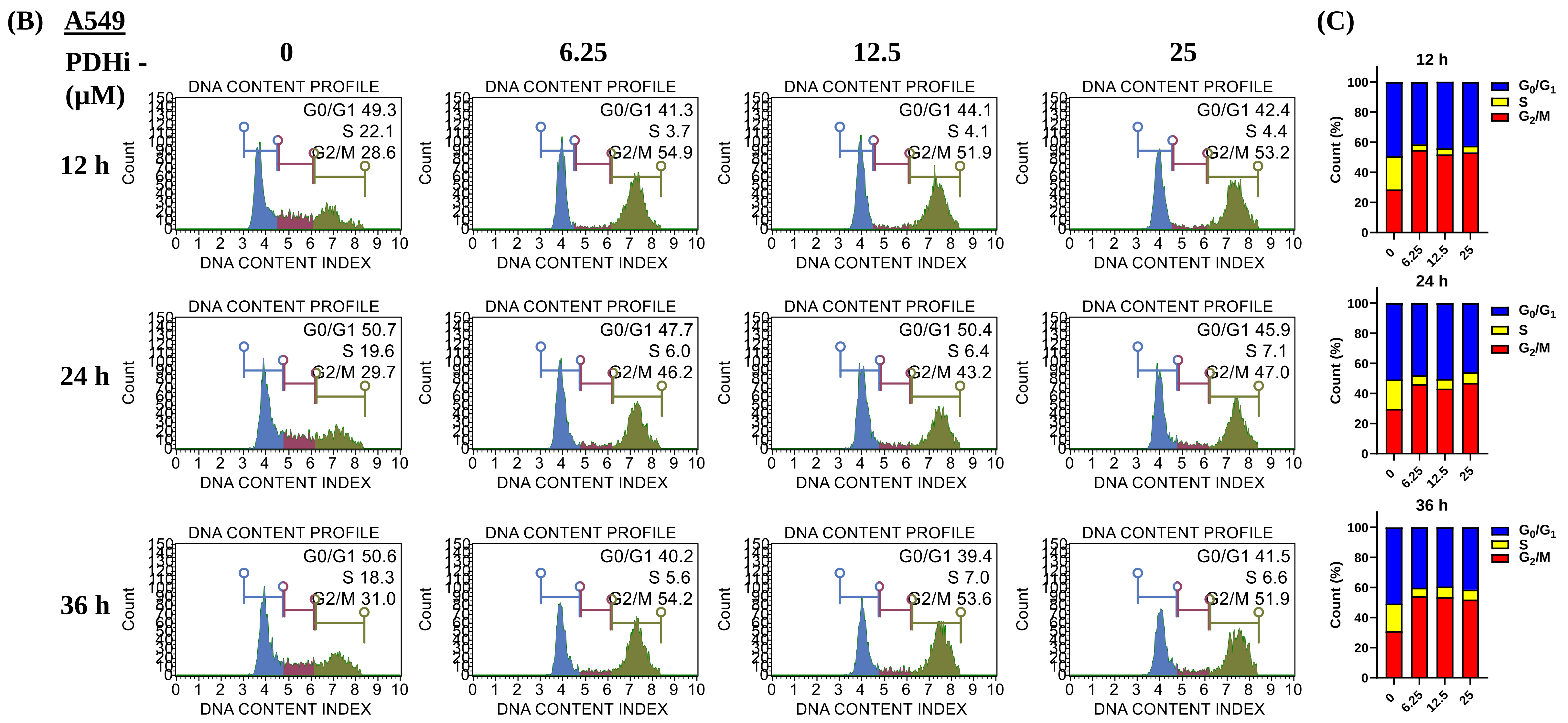

Supplemental Figure 5B and C

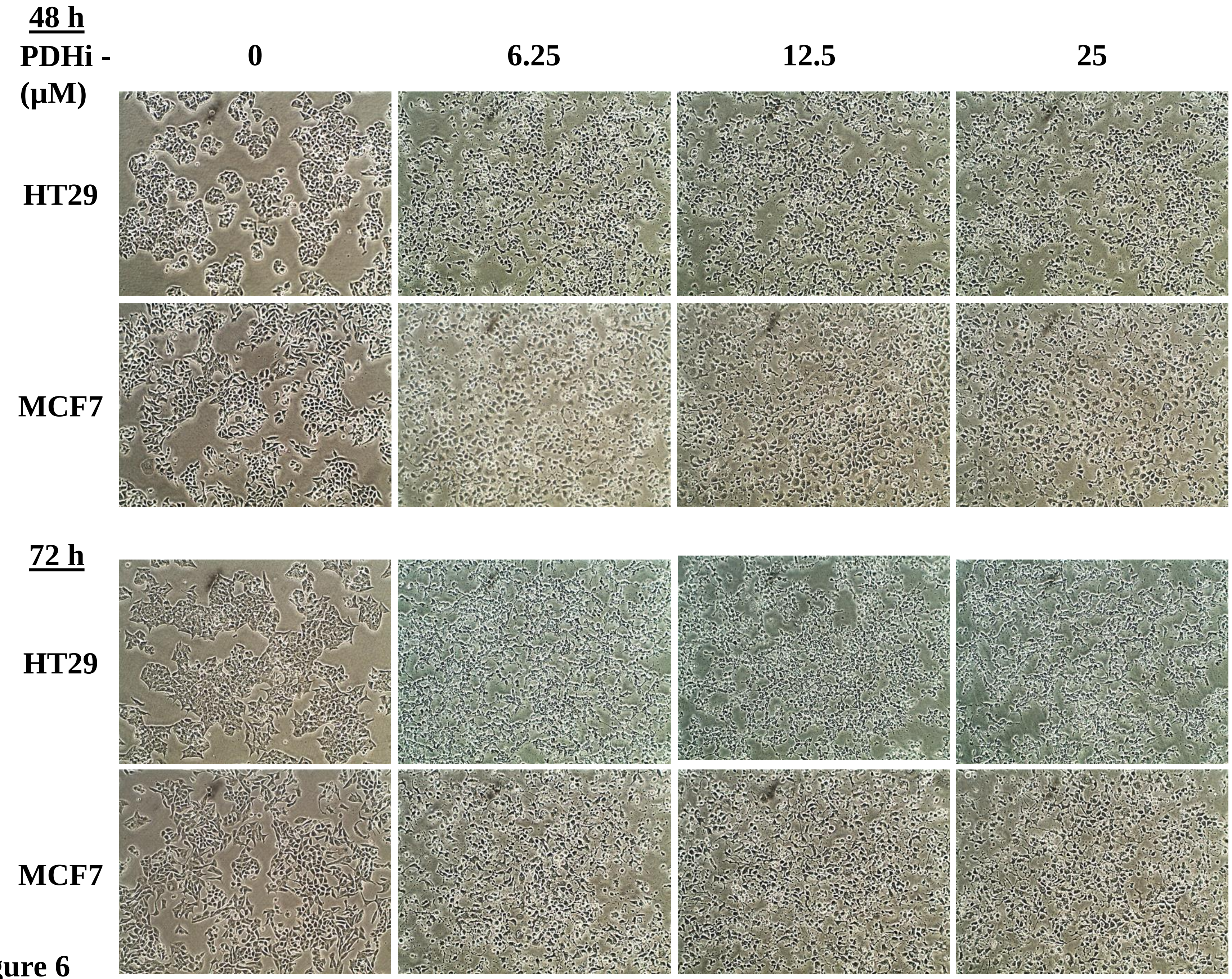

Supplemental Figure 6

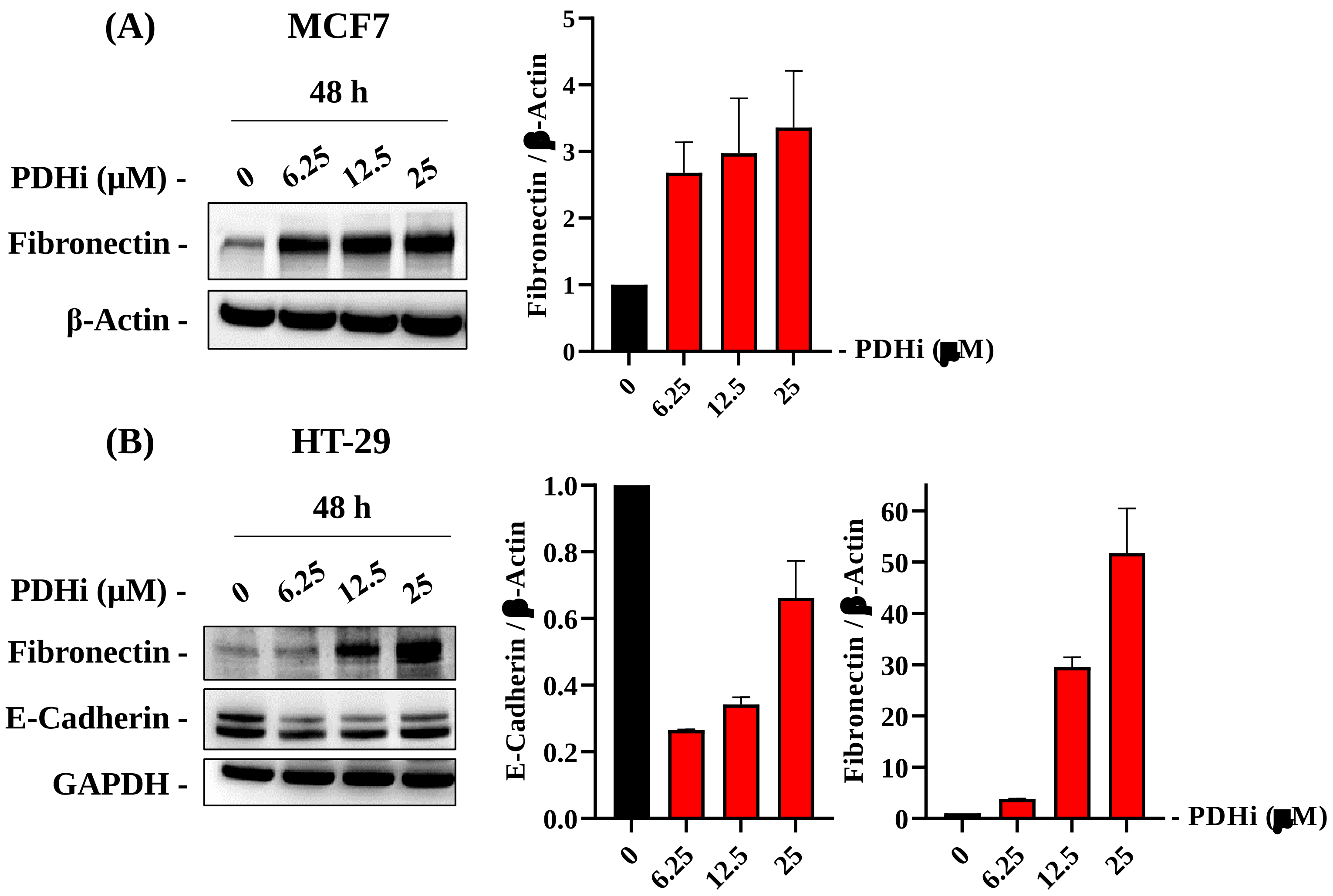

Supplemental Figure 7

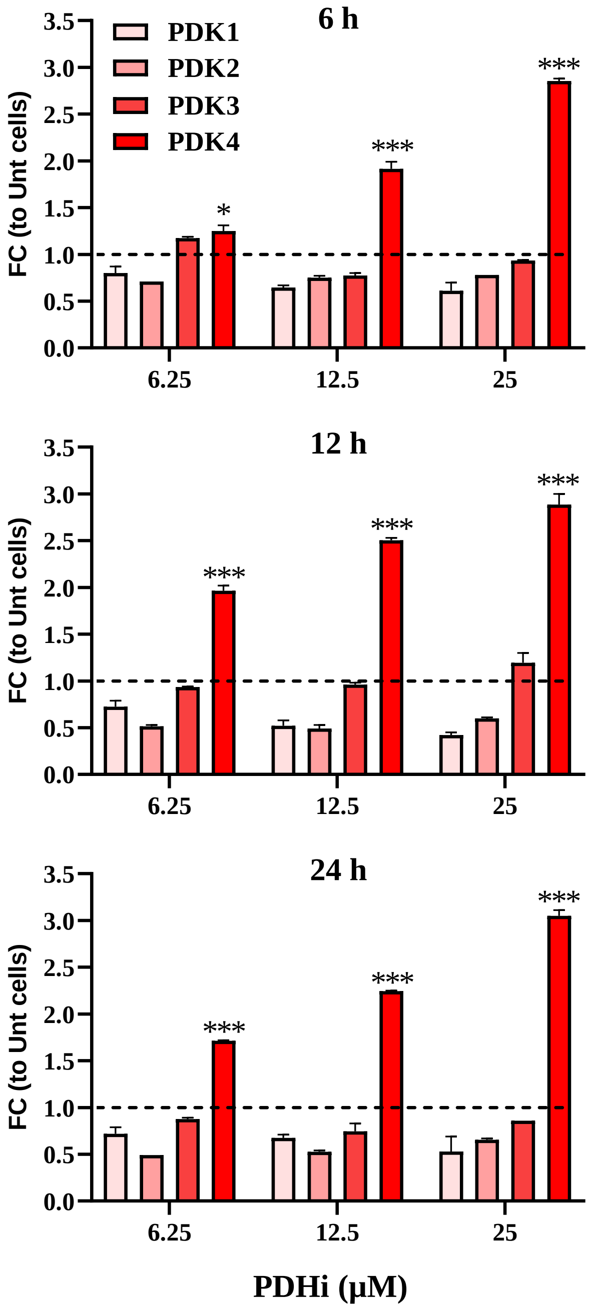

Supplemental Figure 8

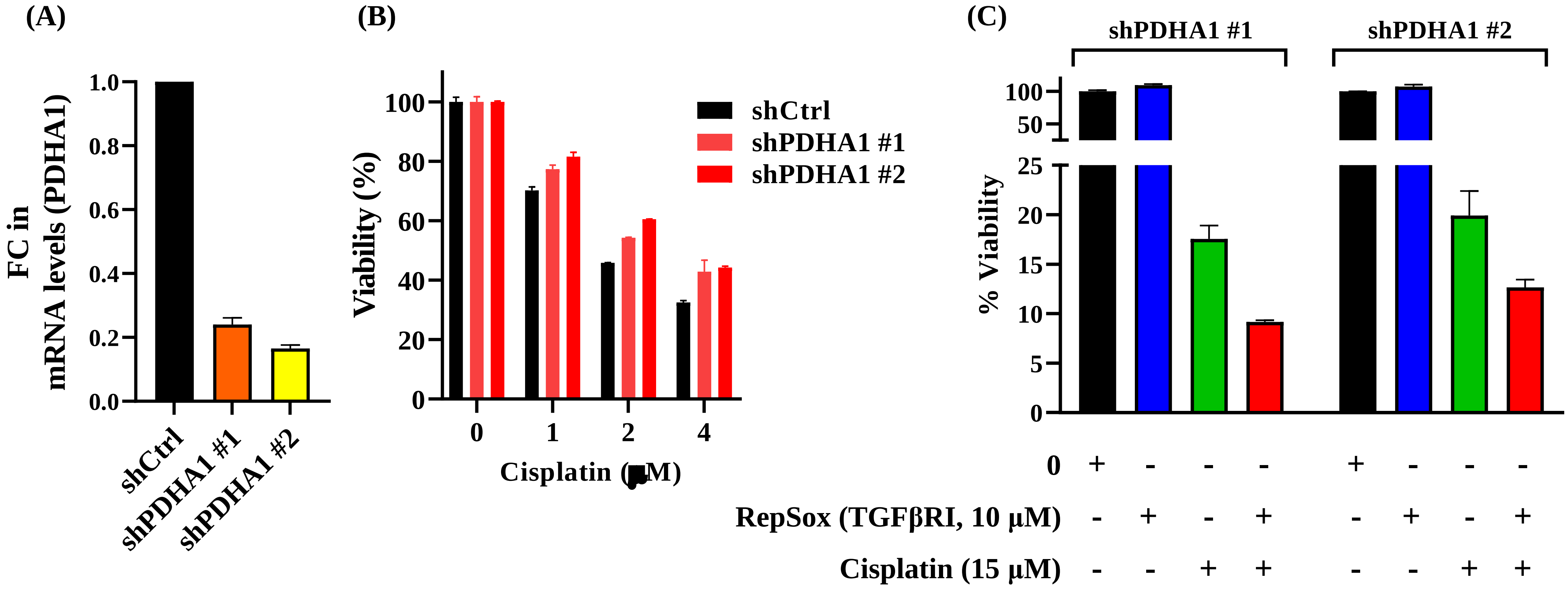

Supplemental Figure 9

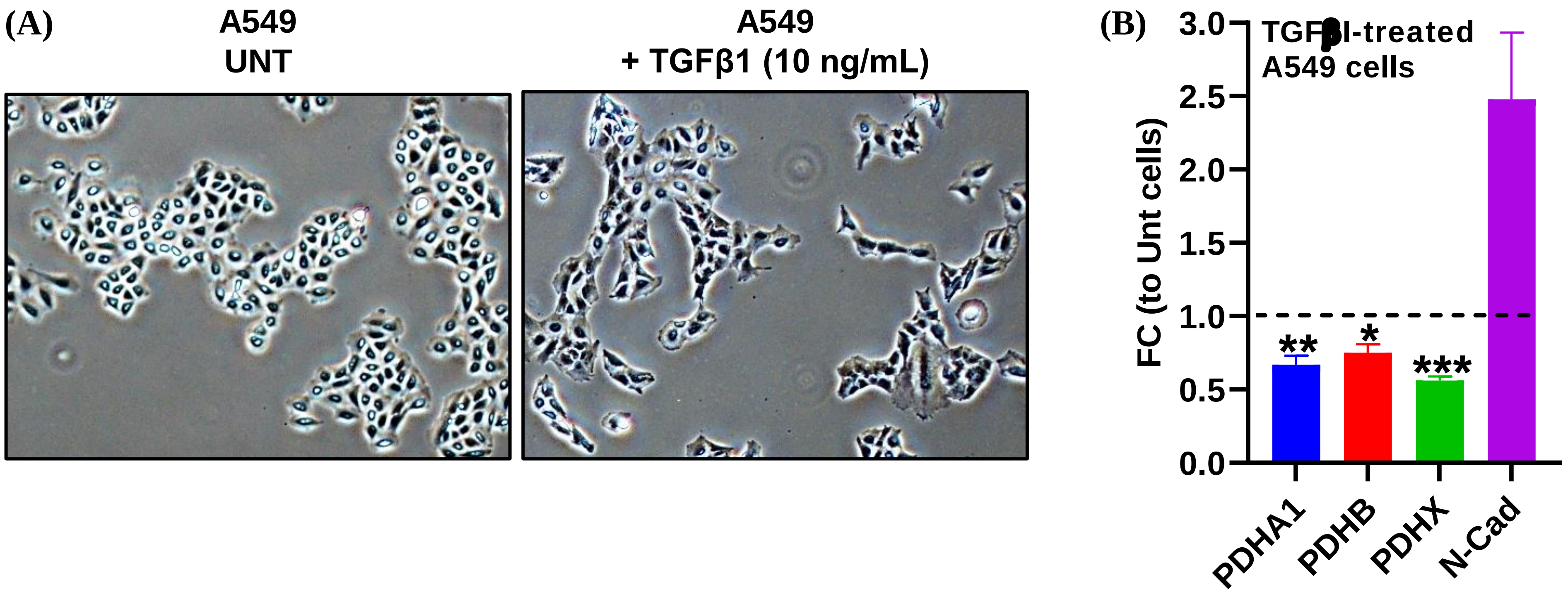

Supplemental Figure 10

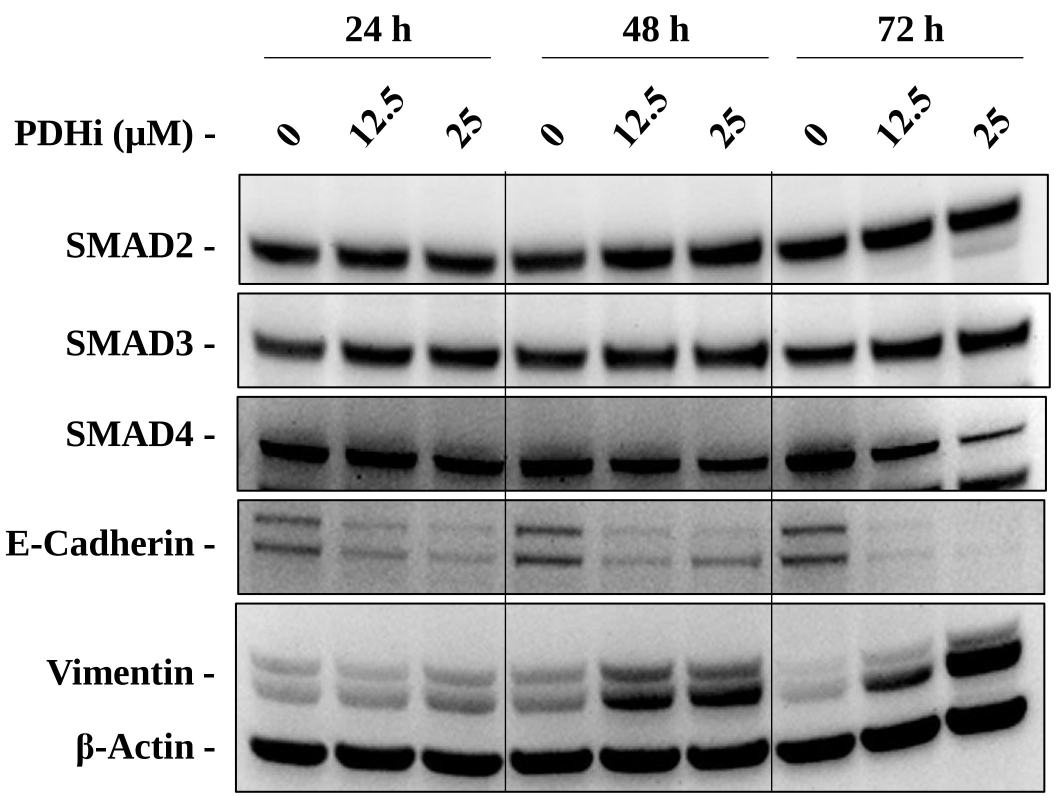

Supplemental Figure 11

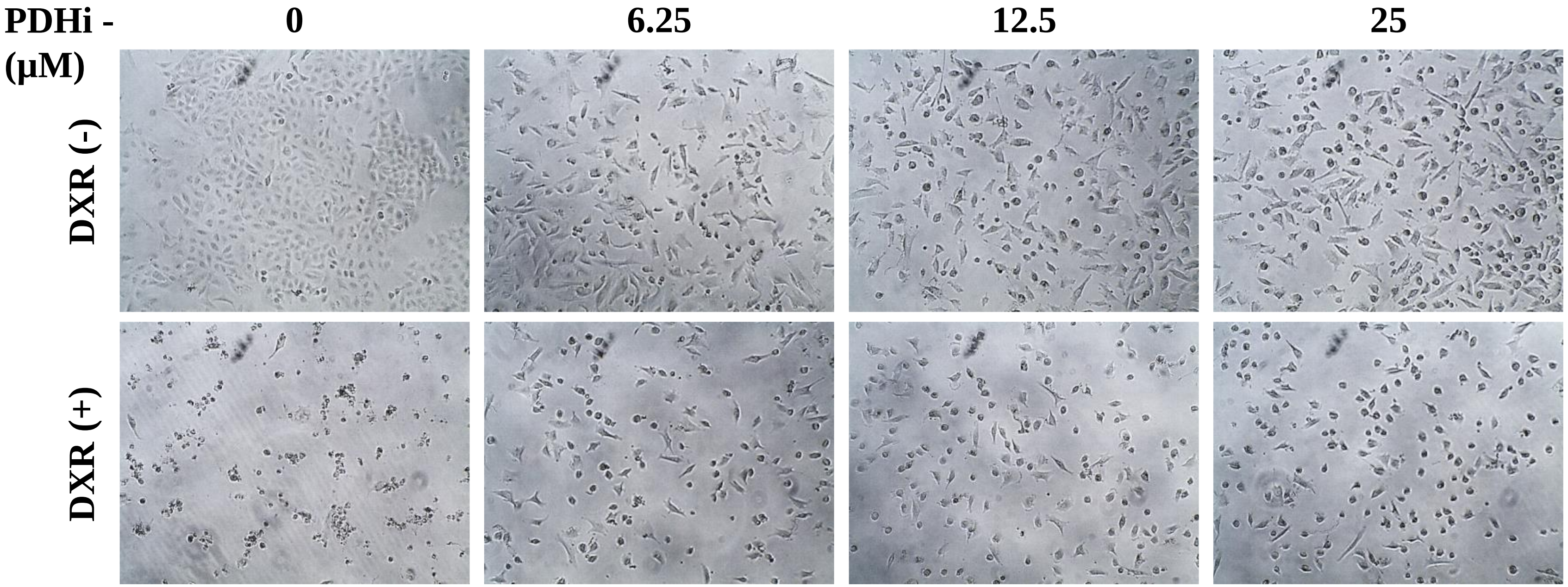

Supplemental Figure 12

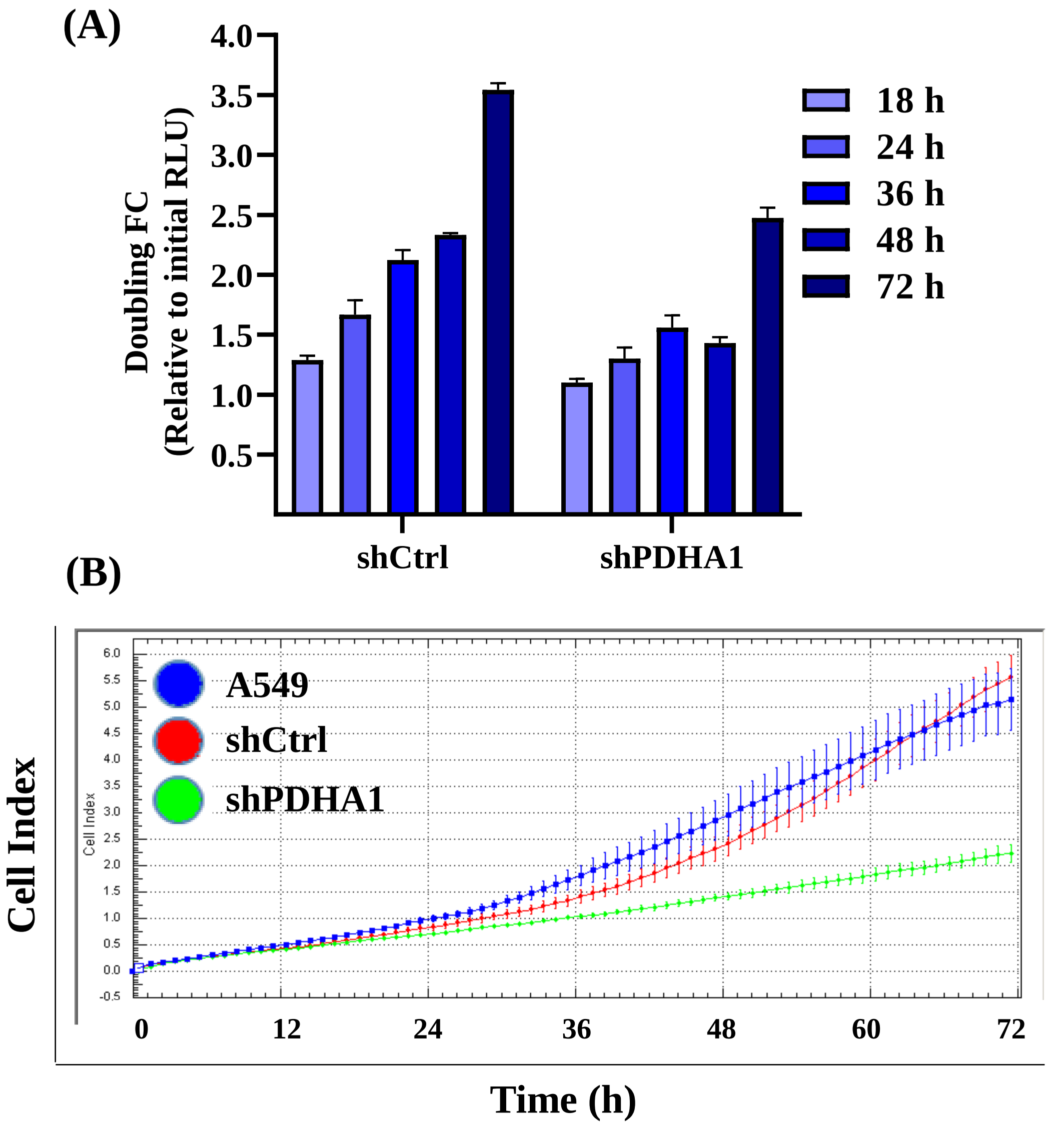

Supplemental Figure 13

Supplemental Figure Captions (I)

Figure 1. PDHi treatment resulted in growth inhibition. (A) A549 cells were treated with PDHi at indicated doses for 24 h and 48 h and after staining with SRB dye, cells were photographed under phase contrast microscope (magnification 100×) **(B)** Real time analysis of anti-growth effect. The cell index (CI) values for 6.25-200 µM of PDHi on A549 cells are shown. The concentrations represented by different colours.

Figure 2. PDHi treatment resulted in growth inhibition. (A) MCF7 cells were treated with PDHi at indicated doses for 24-72 h and after staining with SRB dye, cells were photographed under phase contrast microscope (magnification 100×) **(B)** The effect of PDHi on the proliferation of MCF7 cells was quantified by SRB and ATP viability assays. Data are presented as mean ± SEM. **(C)** Real time analysis of anti-growth effect. The cell index (CI) values for 6.25-200 µM of PDHi on MCF7 cells are shown. The concentrations represented by different colours. * Significantly difference compared to untreated cells (*$*P < 0.01$, $**P < 0.001$, $***P < 0.0001$*).

Figure 3. PDHi treatment resulted in growth inhibition. (A) HT29 cells were treated with PDHi at indicated doses for 24-72 h and after staining with SRB dye, cells were photographed under phase contrast microscope (magnification 100×) **(B)** The effect of PDHi on the proliferation of HT29 cells was quantified by SRB and ATP viability assays. Data are presented as mean ± SEM. **(C)** Real time analysis of anti-growth effect. The cell index (CI) values for 6.25-200 µM of PDHi on HT29 cells are shown. The concentrations represented by different colours. * Significantly difference compared to untreated cells *($*P < 0.01$, $**P < 0.001$, $***P < 0.0001$).*

Figure 4. PDHi treatment resulted in apoptotic cell death. (A) Cells treated with PDHi for 48 h at indicated doses and were photographed under differential interference contrast (DIC) microscope **(B)** PDHi resulted in chromatin condensation and pyknosis in A549 cells which is a hallmarks of apoptosis **(C)** Plasma membrane binding of Annexin-V was used to detect and quantify apoptotic cells induced by PDHi (24-72 h) in A549 cells **(D)** Pretreatment with the pan-caspase inhibitor (z-VAD-FMK) rescued cell death induced by PDHi. Cell viability (24 h and 48 h) of A549 cells was quantified by ATP assay. Data are presented as mean ± SEM. * Significantly difference compared to z-VAD-FMK (-) cells *($*P < 0.01$, $***P < 0.0001$).*

Figure 5. PDHi treatment resulted in G_2/M cell cycle arrest. (A) Cells treated with PDHi for 12, 24 and 36 h at indicated doses and were photographed under phase contrast microscope. **(B)** Cell cycle histogram of representative results obtained from A549 cells. PDHi treatment increased G_2/M cell accumulation at all doses tested. **(C)** Bar graphs represent the quantified values of the histograms.

Figure 6. PDHi-induced morphological changes in MCF7 and HT29 cancer cells. Cells treated with PDHi for 48-72 h at indicated doses and were photographed under phase contrast microscope (magnification 100×).

Supplemental Figure Captions (II)

Figure 7. PDHi led to the induction of EMT phenotype. (A-B) Western blot analysis showing the decrease of E-cadherin (epithelial marker) and increase in fibronectin (mesenchymal marker) in PDHi treated MCF7 and HT29 cancer cells.

Figure 8. PDHi treatment resulted in PDK4 upregulation. A549 cells treated with PDHi for 6, 12 and 24 h at indicated doses and expression levels of PDK4 mRNA were determined by qRT-PCR. β-Actin was used as reference control. Significantly difference compared to untreated cells *(*P < 0.01, ***P < 0.0001).*

Figure 9. Inhibition of TGFβRI diminishes cisplatin resistance. (A) Knockdown (stable) efficiency of shPDHA1 and the control (shCtrl) vectors in A549 lung cancer cells was determined via qRT-PCR to confirm PDHA1 gene expression. Data are presented as mean ± SEM. **(B)** sh-labeled A549 cells treated with Cisplatin for 72 h at indicated doses. **(C)** These cells were co-incubated with 10 μM of RepSox (TGFβRI-i and cisplatin for 72 h. Growth inhibition (B-C) was analyzed by SRB viability assay. Data are presented as mean ± SEM.

Figure 10. TGFβI treatment decreased the expression levels of PDH genes (PDHA1, PDHB, and PDHX). (A) A549 cells were treated with TGFβI for 72 h (10 ng/ml) and photographed under phase contrast microscope. **(B)** TGFβI-treated A549 cells were subjected to qRT-PCR to determine the transcript levels of PDH genes. β-Actin was used as reference control. Significantly difference compared to untreated cells *(*P < 0.01, **P < 0.001, ***P < 0.0001).*

Figure 11. SMAD proteins (SMAD2, 3 and 4) of canonical TGFβ signaling pathway were immunoblotted upon PDHi treatment. A549 cells treated with PDHi for 24, 48 and 72 h at indicated doses. Blot also shows the EMT-induction upon PDHi treatment (the decrease of E-cadherin and increase in vimentin expression).

Figure 12. PDHi treatment decreased the chemotherapeutic drug sensitivity of A549 cells. PDHi pretreated (6.25, 12, 25 μM) or non-pretreated (0 μM) A549 cells were exposed to Doxorubicin (1.8 μM) for 72 h and photographed under phase contrast microscope (magnification 100×).

Figure 13. The effect of PDHA1 silencing on lung cancer cell growth/proliferation. The doubling of cells was quantified by **(A)** ATP assay and **(B)** Real time analysis.
